# A set of multidimensional indicators to assess the resilience and attractiveness of Italian provinces and municipalities (2010–2022 panel data)

**DOI:** 10.1016/j.dib.2024.111042

**Published:** 2024-10-19

**Authors:** Elsa Amaddeo, Marika Arena, Angela Stefania Bergantino, Giovanni Bonaccorsi, Alessandro Buongiorno, Antonello Clemente, Andrea Flori, Mario Intini, Francesco Scotti, Valeria Maria Urbano, Michele Vitagliano

**Affiliations:** a Department of Economics, Management and Business Law, University of Bari Aldo Moro, Largo Abbazia S. Scolastica, 53-70124 Bari, BA, Italy; b Department of Management, Economics and Industrial Engineering, Politecnico di Milano, Via Raffaele Lambruschini, 4/B, 20156 Milano, MI, Italy

**Keywords:** Economic development, Spatial analysis, Policy evaluation, Socioeconomic factors, Infrastructure analysis, Urban planning

## Abstract

This article provides a panel dataset on four capital dimensions (economic, human, social and physical) to study and promote the attractiveness and resilience of Italian territories.

The dataset is articulated at the provincial and municipal level for the period 2010–2022. Data have been sourced from different open data repositories or collected through scraping downloads and have been elaborated in order to generate novel territorial indicators. While traditional datasets are commonly available at the regional and provincial levels, territorial analyses necessitate more granular data. Hence, this dataset allows researchers to study territorial characteristics of Italy at the NUTS3 and municipal levels, granting different degrees of spatial granularity and potentially supporting policymakers in evaluating the effectiveness of territorial policies implemented over the years.

Specifications Table

**Table:** 

Subject	Economics and Econometrics
Specific subject area	Territorial Analysis, Territorial characteristics, Socio-Economic Analysis, Policy Evaluation.
Type of data	Table, Image, Chart, Graph, Figure, Raw, Processed, Maps
Data collection	The data collection moves, for both spatial levels, from the four dimensions of capital: economic, social, human and physical. All data have been collected from open data sources through scraping processes and have been elaborated with R and Python. Furthermore, starting from these data we created new variables and indicators. In particular, data concerning the economic capital have been collected mainly from the Italian National Institute of Statistics (ISTAT), the European Commission and the Italian Ministry of Economy and Finance. Moving to the human capital dimension, data related to schools and universities, demography and sanitary services have been collected from ISTAT, the Italian Ministry of Education and the Italian Ministry of University and Research. Data concerning the social capital dimension, which refers to civil society and the quality of government such as the electoral turnout and the institutional quality index, have been collected and organized from ISTAT, Eligendo and the Nifo-Vecchione databases. Finally, for the physical capital dimension, data related to the environment, such as emissions, greenery and park coverage and air quality, have been acquired through a scraping process from Urban Index portal, Copernicus and the annual reports published by Legambiente. Data related to housing and households' assets and infrastructure and services have been either downloaded and elaborated from ISTAT (i.e., bike vehicle fleet and number of hotel structures) or collected by scraping from the Urban Index portal (i.e., consumed soil per capita and university presence index).
Data source location	• European Commission: https://commission.europa.eu/statistics_en • EUROSTAT: https://ec.europa.eu/eurostat/data/database • ISTAT: https://www.istat.it • Urban Index: https://www.urbanindex.it/indicatori/indice-di-dinamismo-delle-istituzioni-pubbliche/ • Ministry of Economy and Finance: https://www.mef.gov.it/en/index.html • Ministry of Cultural Heritage: https://www.beniculturali.it/dati-della-cultura • Ministry of Interior: https://elezionistorico.interno.gov.it/eligendohome/opendata.php • Ministry of Education: https://dati.istruzione.it/opendata/opendata/ • Ministry of University and Research: https://ustat.mur.gov.it/ • Nifo-Vecchione: https://sites.google.com/site/institutionalqualityindex/home • Legambiente: https://www.legambiente.it/rapporti-e-osservatori/ • Copernicus: https://www.copernicus.eu/en/access-data/conventional-data-access-hubs • Agcom: https://maps.agcom.it/ • ANFIA: https://www.anfia.it/it/attivita/studi-e-statistiche • ACI: https://www.aci.it/laci/studi-e-ricerche/dati-e-statistiche/open-data.html • Invalsi: https://serviziostatistico.invalsi.it/archivio-dati/?_sft_invalsi_ss_data_collective=open-data
Data accessibility	Repository name: Zenodo Data identification number: 10.5281/zenodo.11121790 Direct URL to data: https://zenodo.org/records/11121791
Related research article	

## 1 Value of the Data

• The dataset tackles the characteristics of Italian territories at both the NUTS3 and municipal levels, granting different degrees of spatial granularity.
• The dataset involves the elaboration of 31 indicators across different territorial dimensions, explaining the capacity of territories to increase their levels of resilience and attractiveness.
• The dataset can allow researchers and policy makers to monitor the attractiveness and resilience of territories over time.
• The dataset can support policy makers in evaluating the effectiveness of territorial policies implemented over time.
• The dataset can be used to answer diverse and interdisciplinary research questions.

## 2 Background

Territorial capacities are defined as the collective capital assets within a community or territory that enhance both its attractiveness and resilience [5] and are often linked to measurable indicators [1]. Several authors have highlighted the connection between attractiveness and resilience, emphasizing the importance of territorial capacities. Territorial attractiveness refers to a territory's ability to draw and retain businesses, investments, skilled labor, and tourists by leveraging its economic, social, environmental, and institutional strengths [8]. On the other hand, territorial resilience is defined as the capacity of a territory to absorb, adapt to, and recover from economic, social, or environmental shocks while maintaining or improving its long-term developmental trajectory [7]. Several studies have approached the determinants of resilience in a holistic manner by adopting the concept of capacities, or territorial capital, which is defined as a system of territorial assets with economic, cultural, social, and environmental components [2,4]. While these definitions may differ slightly, both concepts refer to a set of characteristics—here termed capacities—that promote the attractiveness and resilience of territories.

The capacities that determine territories’ ability to respond to challenges can be measured through a set of indicators, which we have grouped into four main dimensions of capital: economic, human, physical, and social. Our framework builds upon the classification introduced by Camagni et al. [2], which identified four dimensions of territorial capacities: economic, cultural, environmental, and social factors. In contrast, our framework expands the notion of cultural factors to include a broader range of human aspects and extends the concept of environmental assets to encompass more general physical characteristics.

While data on these sets of capital are generally available at the regional and provincial levels, territorial analyses require more granular data. To answer this need, we collect, elaborate and provide 225 variables at the provincial and municipal levels for Italian territories for the period 2010–2022 related to the four dimensions of capital. Additionally, we illustrate the potential use of these data through the construction of composite indicators, that integrate different variables for a unified representation across the four capitals. To conclude, a graphic representation of the indicators is provided.

## 3 Data Description

Building on the definitions of territorial resilience and attractiveness, and their connection to the set of capacities known as territorial capital, a mixed approach combining top-down and bottom-up processes was employed to define relevant indicators and variables. First, a review of existing literature on territorial resilience and attractiveness informed the identification of macro-areas, as outlined in the "Background" section. Next, data collection from open data sources enabled the selection of measurable indicators based on available data. This mixed approach resulted in the identification of key area indicators and the corresponding variables for analysis. This process led to the development of a unique database, incorporating 133 variables at NUTS3 level and 92 at municipal level. The data collection and preprocessing methods, as well as the methodology adopted for developing synthetic indicators is detailed in the section "Experimental design, materials and methods".

Concerning the spatial granularity of the data, the database provides information at the municipal and provincial (NUTS-3) levels. Data have been collected from different data sources. Data at the regional level (NUTS-2) can be obtained through the aggregation of the NUTS-3 data. In particular, at the NUTS3 level, there are 57 variables for the economic capital, 2 for the social capital, 57 for the physical capital and 17 for the human capital; at the municipal level, there are 22 variables for the economic capital, 4 for the social capital, 34 for the physical capital and 32 for the human capital.

The economic capital is represented by the macro-areas economy and labor market, where the former includes variables such as Gross Domestic Product (GDP), available in different versions, Gross Value Added (GVA), disposable income and availability of financial markets, and the latter variables related to the employment.

Social capital is declined in the macro-areas civil society, encompassing variables such as employees in social organizations and elections, and government.

Physical capital contains the highest number of collected variables and can be divided into four principal macro-areas: environment, which includes information about air quality, emissions, water capacity and greenery; geography; housing and households assets, which encompasses data related to transportation access (railways stations, airports, vehicles fleets) and the quality of the housing stock constructions; infrastructures and services, providing information on tourist capacity, access to internet and electricity and cultural capacity.

Human capital includes three macro-areas: demography, where variables related to natality, mortality, migration and population growth are included; education, which gives information about schools and universities; and health, which incorporates variables linked to sanitary services.

**Fig. 1:**
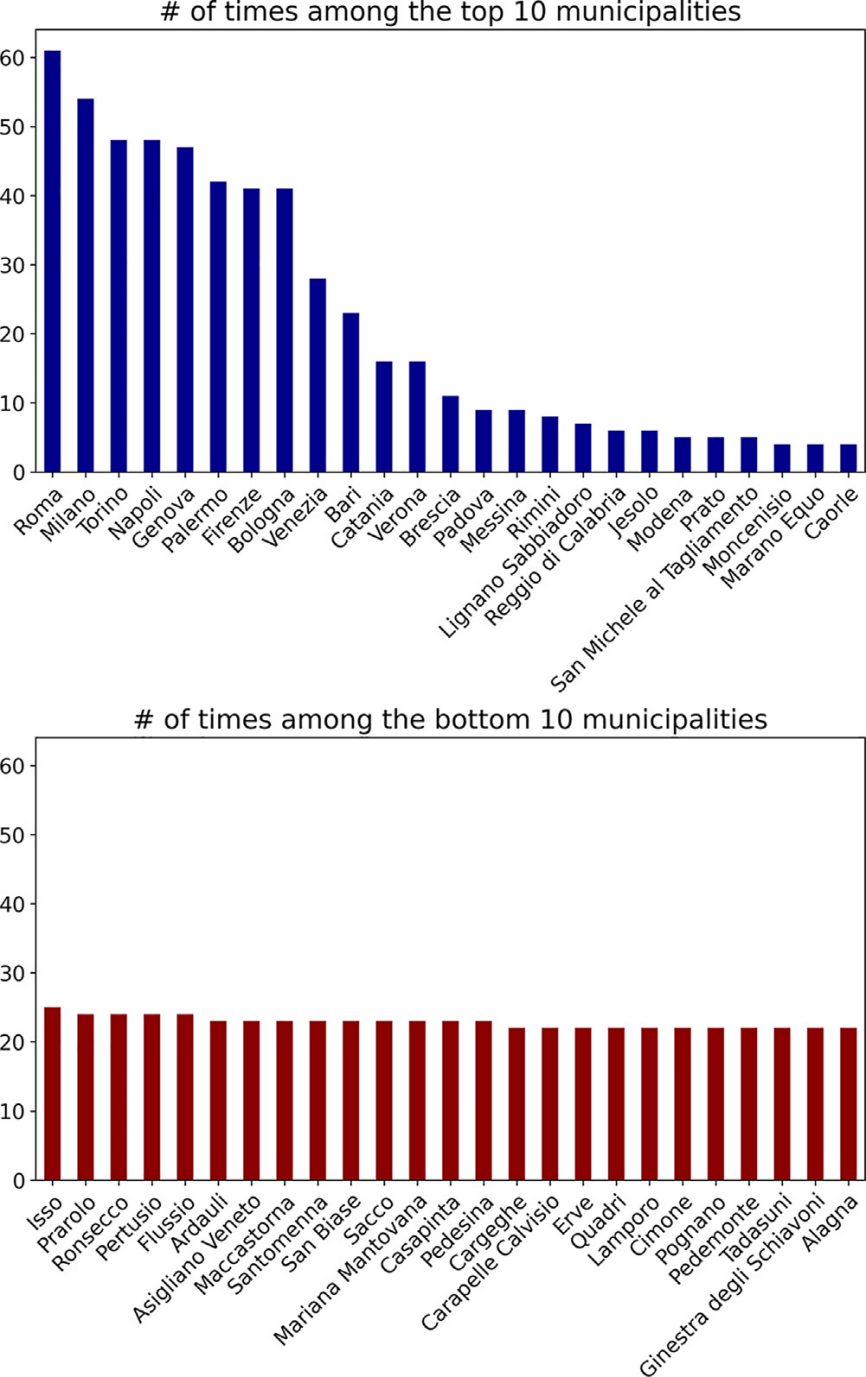
Number of times a certain municipality appears among the top and bottom 10 municipalities for all variables in the dataset. Values shown for the 25 municipalities with the most values. Ranking calculated on data aggregated across all years.

**Fig. 2:**
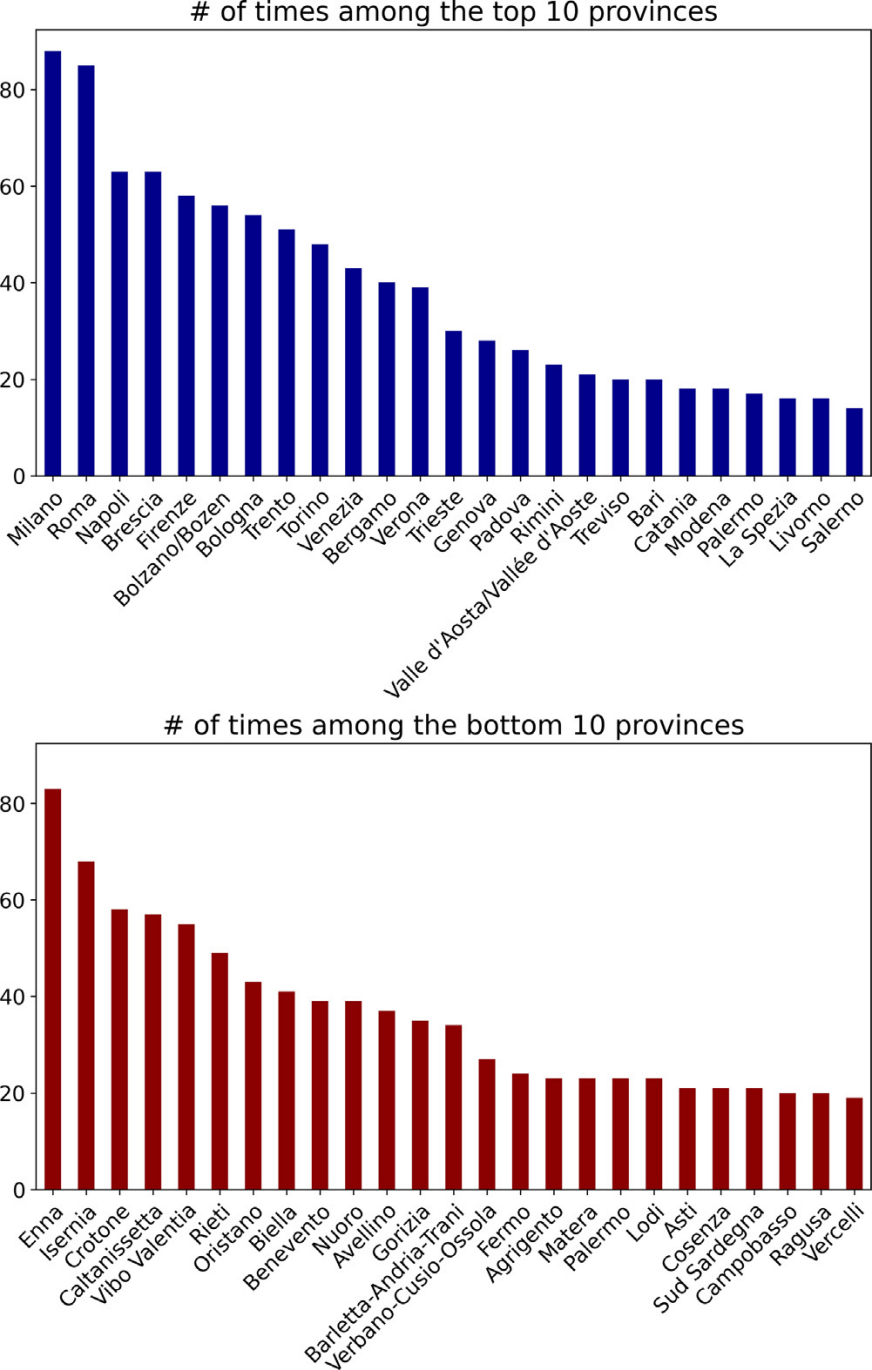
Number of times a certain province appears among the top and bottom 10 provinces for all variables in the dataset. Values shown for the 25 provinces with the most values. Ranking calculated on data aggregated across all years.

To demonstrate the content of the database, Fig. 1, Fig. 2 show the distribution of Italian municipalities and provinces with respect to their performance across different indicators related to the four capital dimensions in the dataset: economic, human, social, and physical. The charts visualize how often certain municipalities and provinces appear among the top 10 (left, blue bars) and bottom 10 (right, red bars) for all measured variables aggregated across all years, from 2010 to 2022.

In Fig. 1 we can observe that a few municipalities, such as Roma and Milano, exceedingly appear in the top rankings. Conversely, municipalities in the bottom 10 positions are more equally distributed, appearing on average 20 times. In Fig. 2 we see instead a skewed distribution both in the top 10 and in bottom 10 chart. On the one hand, the province of Milan dominates the top 10 rankings, appearing in the top tier more than 80 times. On the other hand, provinces like Caserta and Crotone appear most frequently among the bottom 10.

In conclusion, these plots demonstrate the extent of the information in our database. Further details regarding the distribution of the variables in the dataset are available in the Supplementary material where we report descriptive statistics and each of the top 3 and bottom 3 territories for all variables in the dataset.

**Fig. 3:**
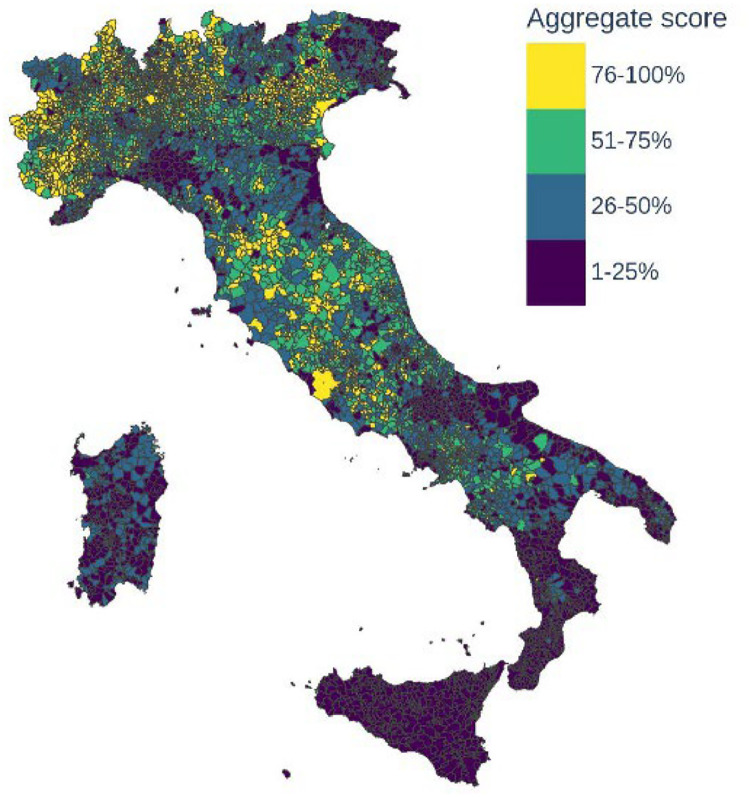
Aggregate score: multidimensional indicator calculated on all dataset dimensions for all municipalities. The score ranges from 1 to 4 and corresponds to the quartiles of the distribution.

**Fig. 4:**
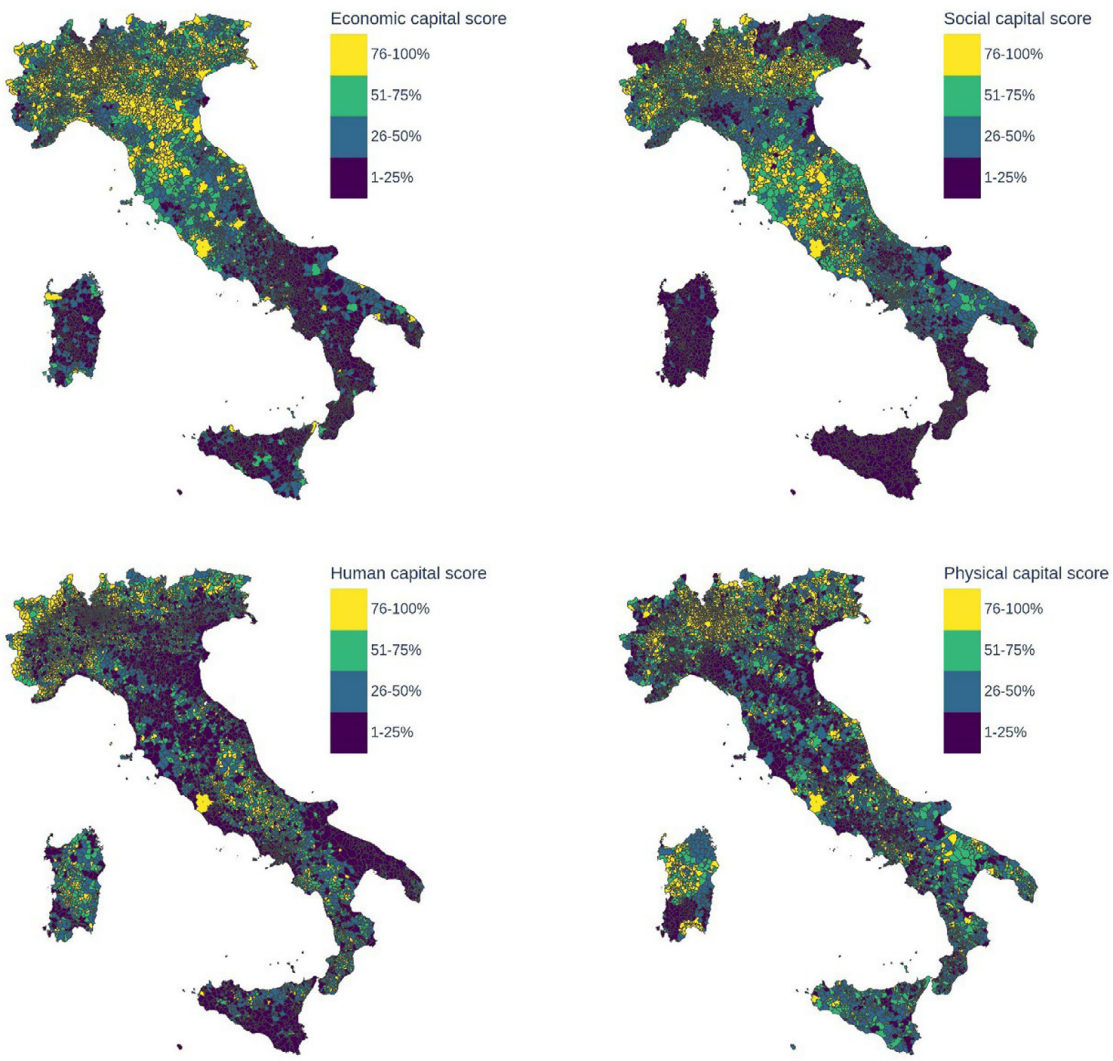
Capital scores: multidimensional indicators obtained for all municipalities from the variables contained in each of the four capital dimensions: economic, social, human, physical. The scores range from 1 to 4 and correspond to the quartiles of the distribution.

Finally, to summarize the information of the multiple features of the dataset we constructed 31 multidimensional indicators using the methodology explained in the section “Experimental design, materials and methods”. These indicators are obtained from the aggregation of the variables at the different hierarchical levels of our database and allow the comparison of territories on the same numerical scale (from 1 to 4, corresponding to the quartiles of the distribution of the indicators) across the different dimensions of the dataset. We plot in Fig. 3, Fig. 4 the multidimensional indicators for all the dataset (aggregate score) and for the four capital dimensions at the municipality level with a colour scale where darker colours correspond to lower levels of the aggregate score.

Table 1 gives a schematic vision of the capitals, macro-areas and areas of the database, while Table A, Table B in the appendix show the main features of all the collected variables and indicators.

**Table 1:** Structure of the four capital dimensions.

Capital	Macroarea	Area	Territorial dimension
Economic capital	Economy	Availability of financial markets	Municipal
		Disposable Income pc	Municipal
		GDP, GVA	NUTS3
	Labour market	Employment Primary/Secondary/tertiary Industry proportion	Both NUTS3
Human capital	Demography	Population Age Dependence Growth Migration Mortality Natality	Both NUTS3 NUTS3 NUTS3 Both Both NUTS3
	Education	University	Municipal
	Health	Health services	Municipal
Physical capital	Environment Geography	Emissions	Both
		Greenery/park coverage Air quality	Both NUTS3
		Water capacity Precipitation	Municipal NUTS3
	Housing and household assets	Housing stock construction quality	Both
		Transportation access	Both
	Infrastructure and services	Density of water supply	Municipal
		Tourist capacity	Both
		Urban road per capita	Municipal
		Cultural capacity Access to electricity, internet	NUTS3 NUTS3
Social capital	Ethnic integration	Foreign population	Both
	Political participation Civil Society Government	Voter turnout Employees in social organizations Government quality	Municipal NUTS3 NUTS3

*Note:* The territorial dimension represents the geographic level at which indicators are exclusively available. In fact, it is always possible to aggregate municipal indicators at the NUTS3 level.

In particular, with reference to Table A, Table B, for each variable we specify: the name under which the variable can be found in the database (label), the area, macro area and capital dimension to which it belongs, the time span for which data are available, the sources and possible notes. Table C shows a list of the 31 indicators and their related capital dimensions.

As shown in Table 2*,* the final database consists of 3 CSV files: one containing all variables collected at the Italian NUTS-3 level (NUTS_3_DATA.csv), another containing those collected at the Italian municipal level (MUNICIPAL_DATA.csv) and a third containing the list of indicators with their scores (INDICATORS.csv).

**Table 2:** Overview of the repository's content. Data file description.

File name	Name of the data in article	N. of the variables	File description
NUTS_3_DATA.csv	TABLE A	133	Data collected at the provincial level or, in some instances, at the level of provincial capital.
MUNICIPAL_DATA.csv	TABLE B	92	Data collected at the municipal level.
INDICATORS.csv	TABLE C	31	Indicators based on the relative scores calculated on the municipal data.

## 4 Experimental Design, Materials and Methods

Since multidimensional datasets at the municipal level are more challenging to obtain, the following sections will focus on delineating the steps to replicate our analysis at this geographical level.

**Fig. 5:**
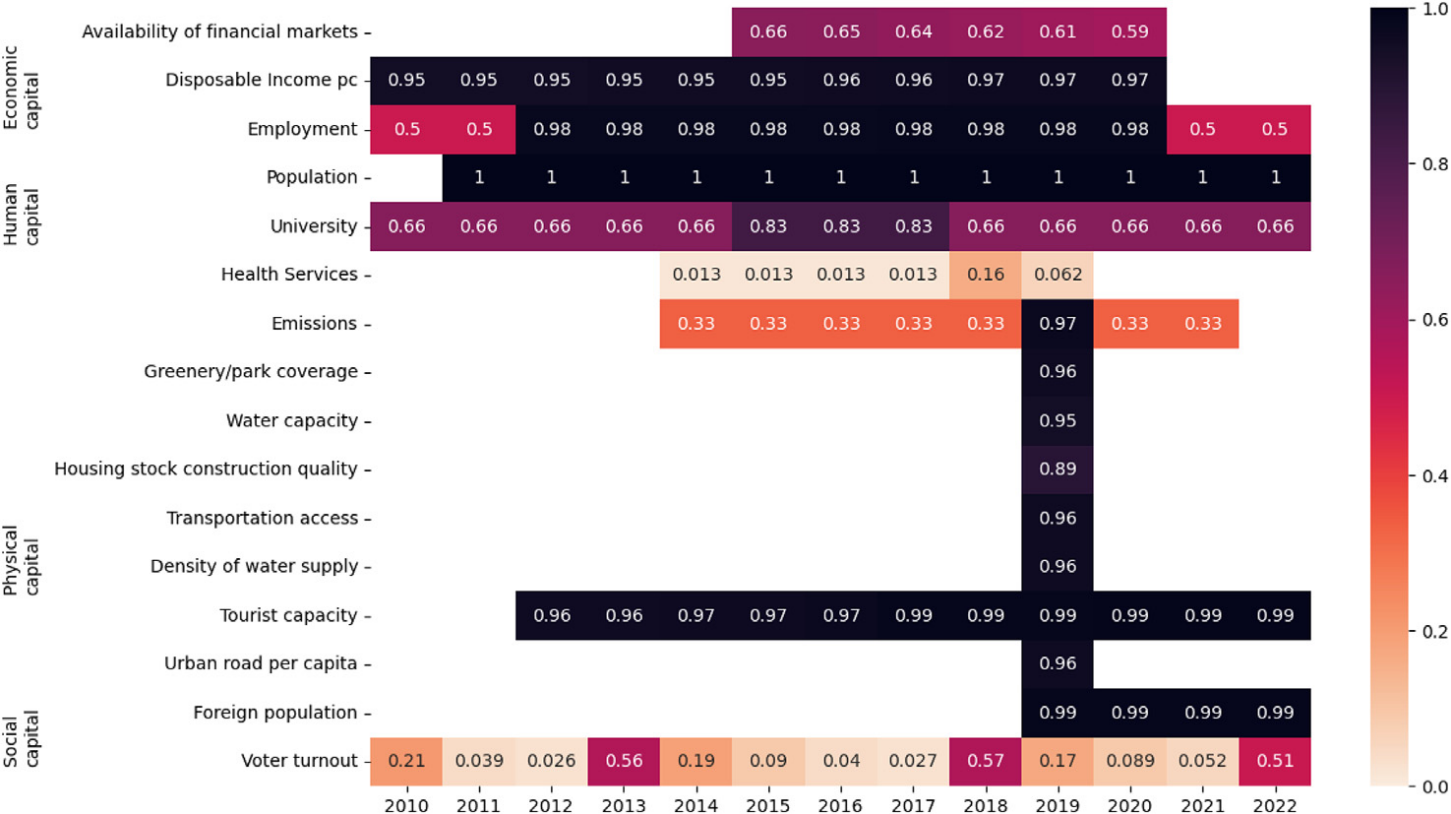
Average percentage of non-missing observations at the municipal level and in each year for the different areas in the database (see Table 1 for details). Each square in the heatmap reports the percentage with a color scale that maps to the reported value. White cells show parts of the of the dataset where information is missing.

**Fig. 6:**
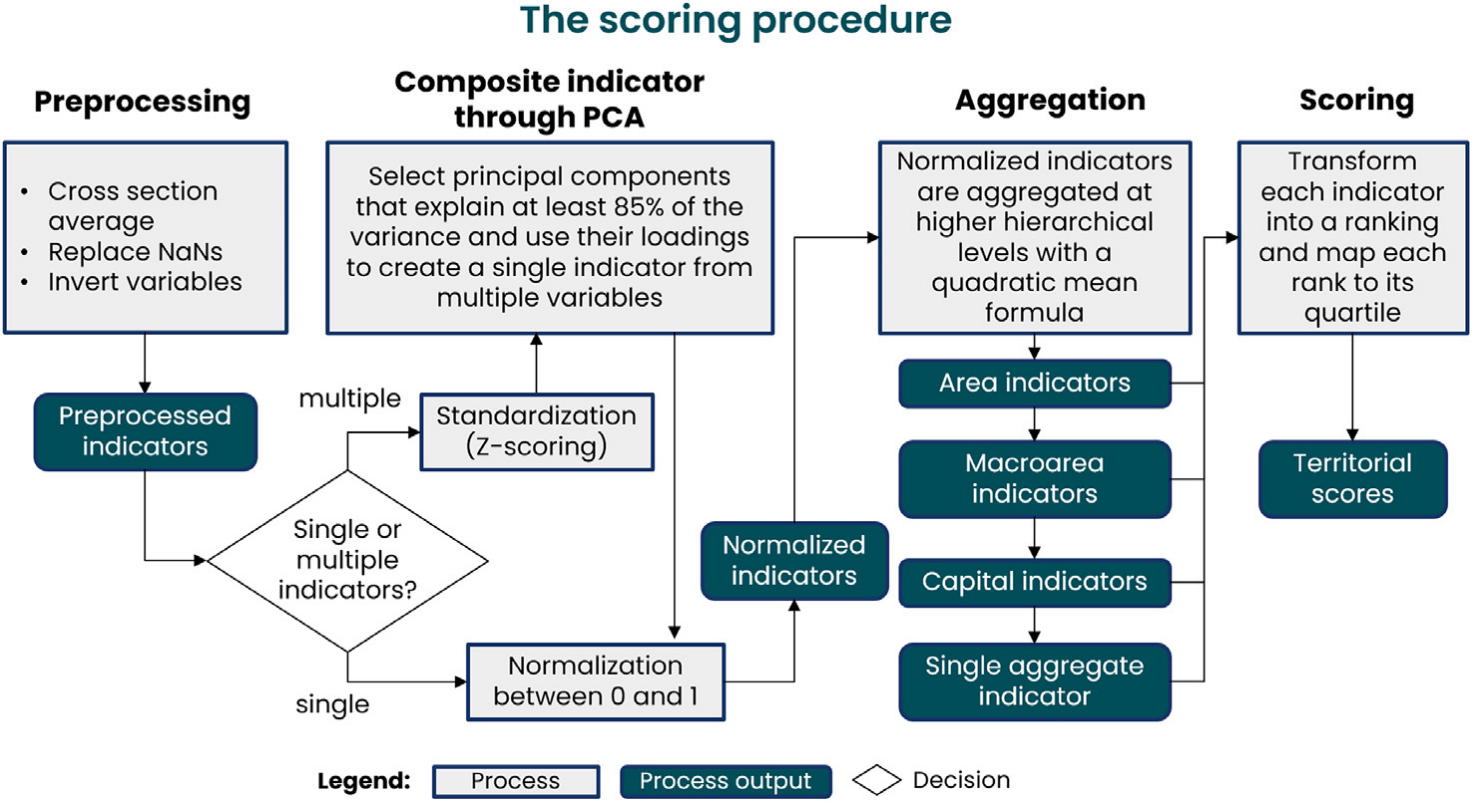
The workflow behind the scoring procedure.

The database construction has followed three main phases. In the first data collection phase, data were gathered from various sources and merged at the appropriate territorial level resulting in a dynamic dataset spanning the years 2010 to 2022 (see Fig. 5). The second phase involved pre-processing the data to homogenize them. This included applying different imputation strategies to remove missing observations, aggregating the data over time, and, finally, inverting variables to align their directions uniformly (higher values indicate greater resilience and attractiveness). Thirdly, in the scoring phase, we processed the data to obtain aggregate scores to compare territories across different dimensions. Fig. 6 shows the steps involved in the second and third phases of the analysis.

### 4.1 Data collection and integration

The data were collected with two different approaches. The first approach entailed downloading the data from different open sources, such as European Commission, EUROSTAT, ISTAT, Urban Index, Ministry of Economy and Finance, Ministry of Cultural Heritage, and Legambiente. Except for PM2.5 emissions data, all the other indicators were geolocalized at the municipal level. This allowed us to merge them seamlessly, obtaining a panel dataset. For PM2.5 emissions we obtained data rasterized on a 1 km × 1 km grid and we proceeded to redistribute the data at a more aggregate level, integrating them with the rest of the indicators, using an Area-Weighted Interpolation methodology [6].

Finally, we constructed aggregated indicators for variables such as the number of workers per sector and the number of enrolled students by degree, in order to capture their primary content in a synthetic manner. After this, we are left with 63 variables in the dataset to use for the construction of multidimensional indicators.

### 4.2 Preprocessing

The dataset obtained from the data collection and integration phases spans from 2010 to 2022, encompassing 7785 municipal units and a total of 63 indicators. In Fig. 5, we aggregate across all municipalities and indicators in the same area, as reported in the third column of Table 1, the average percentage of non-missing observations, leaving the cell empty wherever information is not available. The figure shows that the coverage of indicators is not uniform across time and space. Therefore, we applied the following imputation strategies.

Firstly, the dataset spans several years for many indicators, but it exhibits a different value concentration over time. Therefore, we opted to narrow the analysis on a uniform cross-section of years spanning from 2015 to 2019, thereby excluding years affected by the COVID-19 crisis. Consequently, we aggregated indicators to replace missing values in the resultant dataset, computing averages across the five years of observations from 2015 to 2019. This is equivalent to assuming that, in the five years considered, the territorial values have remained relatively stable with respect to their five-year average, which is a reasonable assumption based on the collected data. It is worth highlighting that, while we are employing an aggregate representation in this work, the data provided cover multiple years, offering a dynamic resource for future research endeavors.

Secondly, to address data gaps we have replaced missing values using the average value of the indicator in the municipalities in the same NUTS3 area for a limited number of indicators reported in the right column of Table 3. Finally, for the remaining indicators, missing values were replaced with zeros. This choice was made coherently with the interpretation of the variables; where the missing data could reasonably indicate a null value, they were replaced with zero. In the remaining cases, the average within the NUTS3 area was utilized.

**Table 3:** Preprocessing for specific variables. Left column: Indicators with inverted value scale. Right column: indicators requiring NUTS3 area average imputation.

Inversion of variables	Imputation of NUTS3 means
Internal_foreigners_migratory_balance	income_pc
Foreigners_census_balance	Deposits
Emergency_unit_distance	Loans
Deaths_total	Employees by sector _total
Deaths_men	emergency_unit_distance
Deaths_women	pm_25
Urban_waste_pc	
Pm_25	
High_hydraulic_hazard_areas_percent	
Population_high_hydraulic_hazard_areas	
Consumed_soil_high_hydraulic_hazard_areas	
Incidence_population_nuclei_scattered_houses	
Housing_dispersion_index	
Private_mobility	
Urban_landscape_fragmentation_index	
Overcrowded_population_percent	
Consumed_soil_pc	

As the last step of the preprocessing procedure, to ensure a consistent interpretation across all variables, we inverted the value scale for indicators with a negative impact, i.e., those for which a higher value implies a less desirable outcome, indicating lower resilience or attractiveness for a territory. We report these variables in the left column of Table 3.

For each of these variables to invert, we use the following formula. Let $x_{jc}$be a single observation of the variable to invert c and $x_{c}$ be the entire distribution of the selected variable in the dataset. Then we obtain the inverted observation $x_{jc}^{\prime }$ as follows:

$$x_{jc}^{\prime }=\frac{x_{jc}-\max\left(x_{c}\right)}{\min\left(x_{c}\right)-\max\left(x_{c}\right)}\cdot \left(\max\left(x_{c}\right)+\min\left(x_{c}\right)\right)+\min\left(x_{c}\right)\;$$

### 4.3 Composition, aggregation and scoring procedure

The complexity and variability of the collected data require a robust standardization procedure to ensure the consistent and objective comparison of territories over time. Therefore, we have opted to convert each territorial dimension to a uniform scale through a scoring procedure [3].

The scoring procedure, reported in Fig. 6, differentiates between areas of indicators where single or multiple indicators were collected. Single indicators are normalized between 0 and 1. Multiple indicators are aggregated with the following procedure: first, we standardize their value subtracting their mean value and dividing by their standard deviation; then, we perform a principal component analysis on the standardized values, retaining components accounting for at least 85 % of the variance in the data. We use the factor loadings resulting from the components, rotated using the “varimax” approach, to obtain weights for aggregating multiple indicators into single ones. Finally, the resulting aggregated indicators are normalized between 0 and 1.

To normalize indicators, we use the following formula. Let $x_{jc}$ be a single observation of the variable to normalize c and let $x_{c}$ be the entire distribution of the selected variable in the dataset. Then we obtain the normalized observation ${\tilde{x}}_{jc}$ as follows:

$${\tilde{x}}_{jc}=\frac{x_{jc}-\min\left(x_{c}\right)}{\max\left(x_{c}\right)-\min\left(x_{c}\right)}$$

This procedure enabled us to generate scores ranging from 0 to 1 for all dimensions in our dataset, facilitating meaningful comparisons between territorial performances across several resilience and attractiveness dimensions. However, the number of scores remains rather significant, prompting the need for more synthetic indicators. For this reason, we have further aggregated the lower-level indicators in area and capital indicators following the hierarchy reported in Table 1. We assigned equal weight to each indicator, but we used a quadratic mean formula to highlight differences across values. For each of our territorial observations *j*, we obtain the quadratic mean of all its K indicators, ${{\bar{x}}_{j}}^{q}$, as follows:

$${{\bar{x}}_{j}}^{q}=\sqrt{\frac{1}{K}\;\sum_{i=1}^{K}x_{ij}^{2}}$$

Finally, to obtain the position of each territory in the distribution of each dimension, we transform the scores into rankings and map all the ranks to their respective quartiles. The resulting rankings for aggregated scores are displayed in Fig. 3, Fig. 4.

## Limitations

The first limitation of this dataset stems from the challenges associated with data collection from various sources, which vary in spatial and temporal coverage and exhibit some missing values. The heterogeneity in data sources may introduce inconsistencies in the dataset; while some regions, for instance, may provide comprehensive data, others might offer incomplete information. Moreover, the presence of missing values complicates the data analysis process, necessitating the use of imputation techniques. Consequently, the disparities in data coverage and the issue of missing values can limit the generalizability of the territorial scores, as they may not be equally representative of all the territories considered.

A second issue concerns the varying number of indicators across territorial dimensions and over time. Due to the different availability of indicators, some territories are assessed using multiple indicators, while others rely on just a few. This discrepancy accentuates the impact of outliers in territories with fewer indicators, complicating comparisons across different dimensions. Additionally, the uneven distribution of indicators over time may lead to an imbalance in evaluating territorial resilience and attractiveness evolution.

## Ethics Statement

The authors have read and follow the Data in Brief ethical guidelines for publication and confirm that the current work does not involve any of the following: human subjects, animal experiments, data collection from social media platforms.

## CRediT Author Statement

**Elsa Amaddeo:** Data curation, Writing, Original draft preparation, Writing - Review & Editing. **Marika Arena:** Writing - Review & Editing, Supervision. **Angela Stefania Bergantino:** Writing - Review & Editing, Supervision. **Giovanni Bonaccorsi:** Data curation, Writing, Original draft preparation, Formal analysis, Visualization, Writing - Review & Editing. **Alessandro Buongiorno:** Data curation. **Antonello Clemente:** Data curation, Writing, Original draft preparation, Writing - Review & Editing. **Andrea Flori:** Writing - Review & Editing. **Mario Intini:** Writing - Review & Editing. **Francesco Scotti:** Data curation, Writing - Review & Editing. **Valeria Maria Urbano:** Data curation, Writing - Review & Editing **Michele Vitagliano**: Data curation

## Figures and Tables

**Table A:** Variables description at NUTS-3 level.

Variable	Label	Capital	Macroarea	Area	Time period	Source	Notes
GDP at current price	gdp_curr_p, gdp_pc_curr_p, gdp_pe_curr_p	Economic Capital	Economy	GDP	2010–2022	European Commission	Available total, per capita and per person employed
GDP at constant price (ref. 2015)	gdp_const_p_2015, gdp_pc_const_p_2015, gdp_pe_const_p_2015	Economic Capital	Economy	GVA	2010–2022	European Commission	Available total, per capita and per person employed
GDP in purchaising power standard	gdp_pps, gdp_pc_pps, gdp_pe_pps	Economic Capital	Economy	GVA	2010–2022	European Commission	Available total, per capita and per person employed
GVA at current price	gva_curr_p, gva_curr_p_pc , gva_curr_p_A, gva_curr_p_B_E, gva_curr_p_F, gva_curr_p_G_J,gva_curr_p_K_N, gva_curr_p_O_U	Economic Capital	Economy	GVA	2010–2020	European Commission	Available total, per capita and by sector
GVA at constant price (ref. 2015)	gva_const_p_2015, gva_const_p_2015_pc, gva_const_p_2015_A_pc, gva_const_p_2015_B_E_pc, gva_const_p_2015_F_pc, gva_const_p_2015_G_J_pc, gva_const_p_2015_K_N_pc, gva_const_p_2015_O_U_pc	Economic Capital	Economy	GVA	2010–2020	European Commission	Available total, per capita and per capita by sector
GVA in purchaising power standard	gva_pps, gva_pps_pc, gva_pps_A, gva_pps_B_E, gva_pps_F, gva_pps_G_J, gva_pps_K_N, gva_pps_O_U,	Economic Capital	Economy	GVA	2010–2020	European Commission	Available total, per capita and by sector
GDP growth rate	gdp_g, gdp_pc_growth_current_p, gdp_pc_growth_const_p_2015, gdp_pc_growth_pps	Economic Capital	Economy	GDP growth	2010–2022	European Commission and Research Team Computation	Available total, per capita at current price, per capita at constant price and per capita in purchasing power standards
GVA growth rate	gva_A_growth_pps, gva_B_E_growth_pps, gva_F_growth_pps, gva_G_J_growth_pps, gva_K_N_growth_pps, gva_O_U_growth_pps, gva_pc_growth_pps, gva_pc_growth_pps_A, gva_pc_growth_pps_B_E, gva_pc_growth_pps_F, gva_pc_growth_pps_G_J, gva_pc_growth_pps_K_N, gva_pc_growth_pps_O_U	Economic Capital	Economy	GVA growth	2010–2022	European Commission	Available total by sector, per capita and per capita by sector
Number of Employees	employment_sum	Economic Capital	Labour market	Employment	2010–2022	European Commission	
Number of Employees by sector	employment_A, employment_B_E, employment_F, employment_G_J, employment_K_N, employment_O_U	Economic Capital	Labour market	Primary/Secondary/tertiary Industry proportion	2010–2020	European Commission	Available sectors: A, B-E, F, G-J, K-N, O-U
Weight of cooperative societies	cooperative_societies_weight	Social Capital	Civil Society	Employees in social organizations	2012–2021	ISTAT	
Institutional Quality Index	institutional_quality_index	Social Capital	Government	Government quality	2010–2019	Nifo-Vecchione	
Birth rate per thousand inhabitants	birth_rate_per_thousand	Human Capital	Demography	Natality	2010–2022	ISTAT	
Average number of children per woman	average_children_per_woman	Human Capital	Demography	Natality	2010–2022	ISTAT	
Mean age of childbearing	childbearing_mean_age	Human Capital	Demography	Natality	2010–2022	ISTAT	
Mortality rate per thousand inhabitants	mortality_rate_per_thousand	Human Capital	Demography	Mortality	2010–2022	ISTAT	
Life expectancy - total	life_expectancy_at_birth, life_expectancy_at_65	Human Capital	Demography	Mortality	2010–2022	ISTAT	Available life expectancy at birth and at 65 years
Natural growth per thousand inhabitants	natural_growth_per_thousand	Human Capital	Demography	Growth	2010–2022	ISTAT	
Net migration per thousand inhabitants	internal_net_migration_per_thousand, external_net_migration_per_thousand, total_net_migration_per_thousand	Human Capital	Demography	Migration	2010–2022	ISTAT	Available internal, external and total net migration
Old-age dependency ratio	old_age_dependency_ratio	Human Capital	Demography	Dependence	2010–2022	ISTAT	
Index of structural dependence	structural_dependence_index	Human Capital	Demography	Dependence	2010–2022	ISTAT	
Population on January 1 (%)	population_0_14_years_percent, population_15_64_years_percent, population_65_plus_years_percent	Human Capital	Demography	Age	2010–2022	ISTAT	Available population rate for 0–14 years, 15–64 years and over 65 years
Old-age index on January 1st (percentage)	old_age_index_percent	Human Capital	Demography	Age	2010–2022	ISTAT	
Average age of the population on January 1st	average_population_age	Human Capital	Demography	Age	2010–2022	ISTAT	
PM10	pm_10	Physical Capital	Environment	Air quality	2016–2022	Ecosistema urbano - Legambiente	Mean of annual average values in μg/mc, per capital province
Ozone	ozone	Physical Capital	Environment	Air quality	2016–2022	Ecosistema urbano - Legambiente	Average of the number of days of exceedance of the 8-hour moving average of 120 μg/mc, per capital province
Nitrogen dioxide	nitrogen_dioxide	Physical Capital	Environment	Air quality	2016–2022	Ecosistema urbano - Legambiente	Mean of annual average values in μg/mc, per capital province
Differentiated waste (%)	differentiated_waste	Physical Capital	Environment	Emissions	2016–2022	Original data from Municipalities collected by Legambiente	Percentage of differiated waste on total waste per capital province
Produced waste (kg)	produced_waste	Physical Capital	Environment	Emissions	2016–2022	Original data from Municipalities collected by Legambiente	Chilograms per inhabitant per capital province
Trees/100 inhabitants in publicly owned areas	trees_per_100_inhabitants	Physical Capital	Environment	Greenery/park coverage	2016–2022	Original data from Municipalities collected by Legambiente	
Efficient land use	land_use_efficiency_index	Physical Capital	Environment	Greenery/park coverage	2016–2022	Elab. by Legambiente on ISPRA data	Synthetic index [0–10] of land consumption/residents and level of urbanization/residents per capital province
Consecutive days without rain	days_without_rain	Physical Capital	Geography	Precipitation	2021	ISTAT	
Rent gap between the city center and the outskirts - Capital city	rent_gap_city_center_outskirts	Physical Capital	Housing and household assets	Housing stock construction quality	2021	Real Estate Scenarios	Difference for the average fee of a two -room apartment in the capital municipality - in %.
Vehicle fleet	vehicle_fleet	Physical Capital	Housing and household assets	Transportation access	2019	ANFIA on ACI data	
Expenditure on local public transport (in €)	local_public_transport_expenditure	Physical Capital	Housing and household assets	Transportation access	2021	Aggregated Municipal Budget Items by Province	
Per capita expenditure on local public transport (in €)	local_ public_transport_expenditure_pc	Physical Capital	Housing and household assets	Transportation access	2022	Aggregated Municipal Budget Items by Province	
Bike paths	bike_paths	Physical Capital	Housing and household assets	Transportation access	2017 and 2022	ACI - Automobile Club d'Italia	
Expenditure on roads and road infrastructures (in €)	roads_infrastructure_expenditure, roads_infrastructure_expenditure_pc	Physical Capital	Housing and household assets	Transportation access	2021	Aggregated Municipal Budget Items by Province	Available total and per capita
Vehicle fleet	vehicle_fleet_units	Physical Capital	Housing and household assets	Transportation access	2010–2020	ISTAT	
Number of railway stations	railway_stations_units	Physical Capital	Housing and household assets	Transportation access	2010–2020	ISTAT	Not available for 2016, 2017 and 2018
Number of airports	airports_units	Physical Capital	Housing and household assets	Transportation access	2010–2020	ISTAT	
Number of aircraft takeoffs and landings at airports	aircraft_takeoffs_landings_total, international_flights_takeoffs_landings	Physical Capital	Housing and household assets	Transportation access	2010–2020	ISTAT	Avaliable for total and international flights
Number of arriving and departing passengers at airports	passengers_airports, international_passengers_airports	Physical Capital	Housing and household assets	Transportation access	2010–2020	ISTAT	Avaliable for total and international flights
Tonnage of cargo (mail+goods) loaded and unloaded at airports	cargo_airports_tons	Physical Capital	Housing and household assets	Transportation access	2010–2020	ISTAT	
Number of ships arriving at ports	ships_ports_units	Physical Capital	Housing and household assets	Transportation access	2010–2020	ISTAT	
Tonnage of ships arriving at ports	ships_ports_tonnage	Physical Capital	Housing and household assets	Transportation access	2010–2020	ISTAT	
Tonnage of goods loaded and unloaded at ports	goods_ports_tons	Physical Capital	Housing and household assets	Transportation access	2010–2020	ISTAT	
Number of passengers embarked and disembarked at ports	passengers_ports_units	Physical Capital	Housing and household assets	Transportation access	2010–2020	ISTAT	
Households reached by broadband (FttC >100mbps) (%)	broadband_connectivity_percent	Physical Capital	Infrastructure and services	Access to electricity, internet	2021	Agcom	
Total final consumption of natural gas and electricity in capital cities per 100 inhabitants	energy_consumption_per_100_inhabitants	Physical Capital	Infrastructure and services	Access to electricity, internet	2022	ISTAT	
Households with active broadband lines (%)	broadband_penetration_percent	Physical Capital	Infrastructure and services	Access to electricity, internet	2021	Agcom	
Number of hotel establishments up to 24 rooms	hotels_up_to_24_rooms, hotels_25_99_rooms, hotels_over_100_rooms	Physical Capital	Infrastructure and services	Tourist capacity	2017–2022	ISTAT	Available for hotels with 1–24 rooms, 25–99 rooms and over 100 rooms
Total number of hotel establishments	total_hotel_establishments	Physical Capital	Infrastructure and services	Tourist capacity	2017–2022	ISTAT	
Total World arrivals in establishments	world_arrivals_total, world_arrivals_hotels, world_arrivals_non_hotels	Physical Capital	Infrastructure and services	Tourist capacity	2016–2022	ISTAT	Available for total establishments, hotel establishments and non-hotel establishments
Total arrivals from Foreign Countries in establishments	foreign_arrivals_total, foreign_arrivals_hotels, foreign_arrivals_non_hotels	Physical Capital	Infrastructure and services	Tourist capacity	2016–2022	ISTAT	Available for total establishments, hotel establishments and non-hotel establishments
Total arrivals from Italy in establishments	italy_arrivals_total, italy_arrivals_hotels, italy_arrivals_non_hotels	Physical Capital	Infrastructure and services	Tourist capacity	2016–2022	ISTAT	Available for total establishments, hotel establishments and non-hotel establishments
Total World stays in establishments	world_stays_total, world_stays_hotels, world_stays_non_hotels	Physical Capital	Infrastructure and services	Tourist capacity	2016–2022	ISTAT	Available for total establishments, hotel establishments and non-hotel establishments
Total stays from Foreign Countries in establishments	foreign_stays_total, foreign_stays_hotels, foreign_stays_non_hotels	Physical Capital	Infrastructure and services	Tourist capacity	2016–2022	ISTAT	Available for total establishments, hotel establishments and non-hotel establishments
Total stays from Italy in establishments	italy_stays_total, italy_stays_hotels, italy_stays_non_hotels	Physical Capital	Infrastructure and services	Tourist capacity	2016–2022	ISTAT	Available for total establishments, hotel establishments and non-hotel establishments
Index of receptive density	receptive_density_index	Physical Capital	Infrastructure and services	Tourist capacity	2016–2022	ISTAT	Beds per km2
Receptivity index	receptivity_index	Physical Capital	Infrastructure and services	Tourist capacity	2016–2022	ISTAT	Beds per inhabitant
Tourist intensity index	tourist_intensity_index	Physical Capital	Infrastructure and services	Tourist capacity	2016–2022	ISTAT	Stays per inhabitants
Number of Visitors of State Museums, Monuments, and Archaeological Areas	museums_visitors	Physical Capital	Infrastructure and services	Cultural capacity	2010–2021	Ministry of Cultural Heritage	
Gross revenue of State Museums, Monuments, and Archaeological Areas (€)	museums_gross_revenue_services	Physical Capital	Infrastructure and services	Cultural capacity	2010–2021	Ministry of Cultural Heritage	

**Table B:** Variables description at municipal level.

Variable	Label	Capital	Macroarea	Area	Time period	Source	Notes
Income per capita	income_pc	Economic Capital	Economy	Disposable Income pc	2010–2020	Ministry of Economy and Finance	
Bank branches [number]	bank_branches	Economic Capital	Economy	Availability of financial markets	2015–2020	ISTAT	
Deposits [euros]	deposits	Economic Capital	Economy	Availability of financial markets	2015–2020	ISTAT	
Loans [euros]	loans	Economic Capital	Economy	Availability of financial markets	2015–2020	ISTAT	
Number of Employees	employees_total, employees _B, employees _C, employees _D, employees _E, employees_F, employees_G, employees_H, employees_I, employees_J, employees_K, employees_L, employees_M, employees_N, employees_P, employees_Q, employees_R, employees_S	Economic Capital	Labour market	Employment	2012–2020	ISTAT	Available total number and per sector (sectors: B, C, D, E, F, G, H, I, L M, N, P, Q, R, S)
Internal demographic migratory balance of foreigners	internal_foreigners_migratory_balance	Human Capital	Demography	Migration	2019–2022	ISTAT	
Acquisitions of Italian citizenship	italian_citizenship_acquisitions	Human Capital	Demography	Migration	2019–2022	ISTAT	
Foreigners - total census balance	foreigners_census_balance	Human Capital	Demography	Migration	2019–2022	ISTAT	
University enrollees by municipality of residence	university_enrollees_by_residence,italian_university_enrollees_by_residence,university_enrollees_chemical_pharmaceutical_by_residence,university_enrollees_architecture_by_residence,university_enrollees_geo_biological_by_residence,university_enrollees_medical_by_residence,university_enrollees_engineering_by_residence,university_enrollees_agricultural_by_residence,university_enrollees_economic_statistical_by_residence,university_enrollees_political_social_by_residence,university_enrollees_legal_by_residence,university_enrollees_literary_by_residence,university_enrollees_linguistic_by_residence,university_enrollees_teaching_by_residence,university_enrollees_psychological_by_residence,university_enrollees_physical_education_by_residence,university_enrollees_defense_security_by_residence	Human Capital	Education	University	2015–2017	ISTAT	Available number of total enrollees, italian university enrollees and per academic subject
Number of laureates by sex	laureates_man, laureates_female	Human Capital	Education	University	2010–2022	MUR	
Public care institutions	public_care_institutions	Human Capital	Health	Sanitary services	2014–2019	ISTAT	
Accredited private care institutions	private_care_institutions_accredited	Human Capital	Health	Sanitary services	2014–2019	ISTAT	
Ordinary acute care beds	acute_care_beds_ordinary	Human Capital	Health	Sanitary services	2019	ISTAT	
Long-stay ordinary care beds	long_stay_care_beds_ordinary	Human Capital	Health	Sanitary services	2019	ISTAT	
Rehabilitation ordinary care beds	rehabilitation_care_beds_ordinary	Human Capital	Health	Sanitary services	2019	ISTAT	
Total ordinary care beds	care_beds_ordinary_total	Human Capital	Health	Sanitary services	2019	ISTAT	
Distance from nearest emergency care unit	emergency_unit_distance	Human Capital	Health	Sanitary services	2018	ISTAT, Openmap	
Number of deaths tot	deaths_total, deaths_men, deaths_women	Human Capital	Health	Mortality	2011–2022	ISTAT	Available total number of deaths, for men and women
Production of urban waste per capita	urban_waste_pc	Physical Capital	Environment	Emissions	2019	Urban Index: Indicators for Urban Policies	
Percentage of differentiated waste collection	waste_collection_percent	Physical Capital	Environment	Emissions	2019	Urban Index: Indicators for Urban Policies	
Concentration of PM2.5 emissions	pm_25	Physical Capital	Environment	Emissions	2014–2020	Copernicus	
Urban green (non-agricultural) per capita	urban_green_pc	Physical Capital	Environment	Greenery/park coverage	2019	Urban Index: Indicators for Urban Policies	
Percentage of agricultural land used (SAU) on total agricultural land	agricultural_land_percent	Physical Capital	Environment	Greenery/park coverage	2019	Urban Index: Indicators for Urban Policies	
Percentage of municipal area with high hydraulic hazard P3	high_hydraulic_hazard_areas_percent	Physical Capital	Environment	Water capacity	2019	Urban Index: Indicators for Urban Policies	
Resident population at risk in areas with high hydraulic hazard	population_high_hydraulic_hazard_areas	Physical Capital	Environment	Water capacity	2019	Urban Index: Indicators for Urban Policies	
Area of consumed soil in areas with high hydraulic hazard - P3	consumed_soil_high_hydraulic_hazard_areas	Physical Capital	Environment	Water capacity	2019	Urban Index: Indicators for Urban Policies	
Incidence of resident population in nuclei and scattered houses	incidence_population_nuclei_scattered_houses	Physical Capital	Housing and household assets	Housing stock construction quality	2019	Urban Index: Indicators for Urban Policies	
Index of housing dispersion	housing_dispersion_index	Physical Capital	Housing and household assets	Housing stock construction quality	2019	Urban Index: Indicators for Urban Policies	
Index of concentration of building use types	building_concentration_index	Physical Capital	Housing and household assets	Housing stock construction quality	2019	Urban Index: Indicators for Urban Policies	Inverse proxy of functional mix
Building expansion index in centers and inhabited nuclei	building_expansion_index	Physical Capital	Housing and household assets	Housing stock construction quality	2019	Urban Index: Indicators for Urban Policies	
Use of private vehicles	private_mobility	Physical Capital	Housing and household assets	Transportation access	2019	Urban Index: Indicators for Urban Policies	
Daily mobility for study or work	daily_mobility_study_work	Physical Capital	Housing and household assets	Transportation access	2019	Urban Index: Indicators for Urban Policies	
University presence index (universities and branches)	university_presence_index	Physical Capital	Housing and household assets	Transportation access	2019	Urban Index: Indicators for Urban Policies	
Mobility index (commuting for work reasons)	work_commuting_mobility_index	Physical Capital	Housing and household assets	Transportation access	2019	Urban Index: Indicators for Urban Policies	
Self-sufficiency index (commuting for work reasons)	work_commuting_self_sufficiency_index	Physical Capital	Housing and household assets	Transportation access	2019	Urban Index: Indicators for Urban Policies	
Public mobility	public_mobility	Physical Capital	Housing and household assets	Transportation access	2019	Urban Index: Indicators for Urban Policies	
Slow mobility (on foot or by bicycle)	slow_mobility	Physical Capital	Housing and household assets	Transportation access	2019	Urban Index: Indicators for Urban Policies	
Percentage of Euro 5 and Euro 6 cars on total cars	euro_5_and_6_cars_percent	Physical Capital	Housing and household assets	Transportation access	2019	Urban Index: Indicators for Urban Policies	
Potable water introduced into the municipal network per capita	potable_water_pc	Physical Capital	Infrastructure and services	Density of water supply	2019	Urban Index: Indicators for Urban Policies	
Index of compactness of urban areas	urban_areas_compactness_index	Physical Capital	Infrastructure and services	Urban road per capita	2019	Urban Index: Indicators for Urban Policies	
Index of fragmentation of the urban landscape	urban_landscape_fragmentation_index	Physical Capital	Infrastructure and services	Urban road per capita	2019	Urban Index: Indicators for Urban Policies	
Incidence of population in overcrowded conditions	overcrowded_population_percent	Physical Capital	Infrastructure and services	Urban road per capita	2019	Urban Index: Indicators for Urban Policies	
Consumed soil per capita	consumed_soil_pc	Physical Capital	Infrastructure and services	Urban road per capita	2012–2022	Urban Index: Indicators for Urban Policies	
Hotels and similar (number of structures)	hotels_structures	Physical Capital	Infrastructure and services	Tourist capacity	2012–2022	ISTAT	
Hotels and similar (number of beds)	hotels_beds	Physical Capital	Infrastructure and services	Tourist capacity	2012–2022	ISTAT	
Additional facilities (number of structures)	additional_facilities_structures	Physical Capital	Infrastructure and services	Tourist capacity	2012–2022	ISTAT	
Additional facilities (number of beds)	additional_facilities_beds	Physical Capital	Infrastructure and services	Tourist capacity	2012–2022	ISTAT	
Total hotels and additional facilities (number of structures)	total_hotels_facilities_structures	Physical Capital	Infrastructure and services	Tourist capacity	2012–2022	ISTAT	
Total hotels and additional facilities (number of beds)	total_hotels_facilities_beds	Physical Capital	Infrastructure and services	Tourist capacity	2012–2022	ISTAT	
Receptivity index (beds per inhabitants)	receptivity_index	Physical Capital	Infrastructure and services	Tourist capacity	2012–2022	ISTAT	
Index of receptive density (beds per km2)	receptive_density_index	Physical Capital	Infrastructure and services	Tourist capacity	2012–2022	ISTAT	
Electoral turnout at municipal elections	municipal_elections_turnout	Social Capital	Civil Society	Elections	2015–2022	Eligendo	
Electoral turnout at municipal elections_camera	municipal_elections_camera_turnout	Social Capital	Civil Society	Elections	2015–2022	Eligendo	
Electoral turnout at political elections_senato	political_elections_senato_turnout	Social Capital	Civil Society	Elections	2015–2022	Eligendo	
Electoral turnout at regional elections	regional_elections_turnout	Social Capital	Civil Society	Elections	2015–2022	Eligendo	

**Table C:** Indicators and related capital dimensions.

Area Indicators	Capital
Disposable Income per capita	Economic
Availability of Financial Markets	Economic
Employment	Economic
University	Human
Health Services	Human
Emissions	Physical
Greenery/Park coverage	Physical
Water capacity	Physical
Housing Stock Construction Quality	Physical
Transportation Access	Physical
Density of Water Supply	Physical
Urban Road per capita	Physical
Tourist Capacity	Physical
Population	Human
Voter Turnout	Social
Foreign Population	Human
**Macroarea Indicators**	
Demography	Human
Economy	Economic
Education	Human
Environment	Physical
Ethnic Integration	Human
Health	Human
Housing and Household Assets	Physical
Infrastructure and Services	Physical
Labour Market	Economic
Political Participation	Social
**Capital Indicators**	
Economic Capital	Economic
Human Capital	Human
Physical Capital	Physical
Social Capital	Social
**Aggregate Indicator**	
Aggregate Score	All

## Data Availability

ZenodoA set of multidimensional indicators to assess the resilience and attractiveness of Italian provinces and municipalities in the period 2010–2022 (Original data).

## Appendix B Supplementary materials

Image, application 1
