# Supplementary material for: A set of multidimensional indicators to assess the resilience and attractiveness of Italian provinces and municipalities (2010–2022 panel data)
Source: Data Brief. 2024 Oct 19;57:111042. doi: 10.1016/j.dib.2024.111042 (PMC11567131; doi:10.1016/j.dib.2024.111042)
Supplement: Supplementary file 1 [file mmc1.pdf]

Descriptive statistics for the municipal dataset

	COUNT	MEAN	STD	MIN	25%	50%	75%	MAX
INCOME_PC	83262	16742.77	3610.004	5417.283	13949.54	16816.41	19235.57	62173.48
BANK_BRANCHES	30821	5.011	24.928	1	1	2	4	1445
DEPOSITS	29126	234786.3	3762763	0	0	0	110908.8	2.5E+08
LOANS	29123	289544	5816711	0	0	0	97134	3.86E+08
EMPLOYEES_B	11637	18.253	133.108	0	1.87	4.92	12.43	4807.16
EMPLOYEES_C	66932	491.103	1497.882	0	20.737	104.48	448.912	59931.27
EMPLOYEES_D	25314	29.713	218.997	0	1	3	14	8558.59
EMPLOYEES_E	31042	55.836	272.902	0	3.01	11.21	38.038	12115.62
EMPLOYEES_F	68831	174.404	921.506	0	20.36	57	154.43	64811.57
EMPLOYEES_G	68906	437.409	2877.761	0	24.5	85.075	291.688	173712
EMPLOYEES_H	66342	148.124	1507.381	0	4.84	18.335	65.36	88134.51
EMPLOYEES_I	68593	179.613	1429.488	0	13.34	38.97	113.06	104266.7
EMPLOYEES_J	47876	103.774	1789.364	0	2	7	25.032	100382.7
EMPLOYEES_K	56086	89.702	1178.495	0	3.992	11	34.228	70110.05
EMPLOYEES_L	52360	50.512	401.141	0	3	9.45	31.443	20961.82
EMPLOYEES_M	66877	167.802	2140.177	0	7	22	72.65	152586
EMPLOYEES_N	58239	190.411	2421.524	0	3.97	14.94	57.87	156463.2
EMPLOYEES_P	36312	25.67	195.426	0	1	4	13	9271.51
EMPLOYEES_Q	62870	121.486	1011.14	0	5	18.22	61.808	68824.17
EMPLOYEES_R	47244	33.277	304.699	0	2	5.26	17.73	18027.05
EMPLOYEES_S	61260	67.21	442.883	0	4.698	14.97	45.42	28151.66
EMPLOYEES_TOTAL	69650	2140.831	16784.44	0.83	131.37	459.78	1475.238	1051072
INTERNAL_FOREIGNERS_MIGRATORY_BALANCE	31150	0.01	58.548	-4425	-4	0	4	1272
ITALIAN_CITIZENSHIP_ACQUISITIONS	31150	16.423	94.903	0	0	3	12	6579
FOREIGNERS_CENSUS_BALANCE	31150	1.638	227.641	-18346	-1	0	2	21023
UNIVERSITY_ENROLLEES_BY_RESIDENCE	22983	209.305	1351.661	1	21	57	152	95905
ITALIAN_UNIVERSITY_ENROLLEES_BY_RESIDENCE	22973	202.827	1276.808	1	21	56	148	90166
UNIVERSITY_ENROLLEES_CHEMICAL_PHARMACEUTICAL_BY_RESIDENCE	17650	10.983	45.673	1	2	4	9	2684
UNIVERSITY_ENROLLEES_GEO_BIOLOGICAL_BY_RESIDENCE	17716	12.028	62.263	1	2	4	9	3941
UNIVERSITY_ENROLLEES_MEDICAL_BY_RESIDENCE	20210	25.509	145.211	1	3	7	18	9245
UNIVERSITY_ENROLLEES_ENGINEERING_BY_RESIDENCE	20886	30.053	182.026	1	4	9	23	12694
UNIVERSITY_ENROLLEES_ARCHITECTURE_BY_RESIDENCE	17569	12.308	75.641	1	2	4	9	4798
UNIVERSITY_ENROLLEES_AGRICULTURAL_BY_RESIDENCE	16940	8.075	27.261	1	2	3	7	950
UNIVERSITY_ENROLLEES_ECONOMIC_STATISTICAL_BY_RESIDENCE	20788	31.096	197.608	1	3	9	23	13301
UNIVERSITY_ENROLLEES_POLITICAL_SOCIAL_BY_RESIDENCE	20108	21.959	166.539	1	3	6	15	11552
UNIVERSITY_ENROLLEES_LEGAL_BY_RESIDENCE	19760	24.942	155.209	1	3	7	17	10389
UNIVERSITY_ENROLLEES_LITERARY_BY_RESIDENCE	19349	17.883	129.907	1	2	5	13	9241
UNIVERSITY_ENROLLEES_LINGUISTIC_BY_RESIDENCE	18907	15.288	84.031	1	2	5	12	5305
UNIVERSITY_ENROLLEES_TEACHING_BY_RESIDENCE	18591	12.975	59.955	1	2	5	11	3825
UNIVERSITY_ENROLLEES_PSYCHOLOGICAL_BY_RESIDENCE	16728	10.892	70.865	1	1	3	8	4433
UNIVERSITY_ENROLLEES_PHYSICAL_EDUCATION_BY_RESIDENCE	14067	7.337	35.637	1	1	3	6	2115
UNIVERSITY_ENROLLEES_DEFENSE_SECURITY_BY_RESIDENCE	1939	2.409	10.612	1	1	1	2	270
LAUREATES_MAN	100737	16.53	117.39	0	1	4	12	9659
LAUREATES_FEMALE	100737	22.821	146.579	0	2	6	17	12145
PUBLIC_CARE_INSTITUTIONS	2914	1.188	1.875	0	1	1	1	32

PRIVATE_CARE_INSTITUTIONS_ACCREDITED	2280	1.275	2.307	0	0	1	1	32
ACUTE_CARE_BEDS_ORDINARY	2914	1.188	1.875	0	1	1	1	32
LONG_STAY_CARE_BEDS_ORDINARY	567	14.18	32.993	0	0	0	15	302
REHABILITATION_CARE_BEDS_ORDINARY	567	42.769	107.055	0	0	15	50.5	1747
CARE_BEDS_ORDINARY_TOTAL	567	325.028	778.5	0	66	129	308	12187
EMERGENCY_UNIT_DISTANCE	7896	10.756	6.706	0	6.417	9.881	14.272	151.017
DEATHS_WOMEN	94812	42.862	242.813	0	7	15	35	16084
DEATHS_MEN	94812	39.931	213.037	0	7	15	33	14566
DEATHS_TOTAL	94812	82.793	455.664	0	14	30	68	30650
URBAN_WASTE_PC	7578	396.668	175.426	0	286.725	383.35	470.2	4260.7
WASTE_COLLECTION_PERCENT	7578	46.268	23.132	0	27.8	51.7	65.4	100
PM_25	63096	12.636	5.617	0	8.48	11.104	16.81	32.218
URBAN_GREEN_PC	7623	8.937	216.927	0	0	0	0	18099.7
AGRICULTURAL_LAND_PERCENT	7622	75.004	20.683	0	63.6	82.6	91.3	99.9
HIGH_HYDRAULIC_HAZARD_AREAS_PERCENT	7433	4.493	9.827	0	0	0.9	4.1	100
POPULATION_HIGH_HYDRAULIC_HAZARD_AREAS	7433	250.315	1879.142	0	0	4	58	94049
CONSUMED_SOIL_HIGH_HYDRAULIC_HAZARD_AREAS	7583	11.385	55.365	0	0	1	5	1989
INCIDENCE_POPULATION_NUCLEI_SCATTERED_HOUSES	5372	19.281	18.753	0	4.7	13.3	28.4	97.4
HOUSING_DISPERSION_INDEX	7623	0.104	0.123	0	0.02	0.06	0.14	0.97
BUILDING_CONCENTRATION_INDEX	7623	7491.774	1180.236	604.6	6870.1	7638.8	8286.85	10000
BUILDING_EXPANSION_INDEX	7623	7.976	7.316	0	2.5	5.9	11.6	66.4
PRIVATE_MOBILITY	7623	65.417	9.552	8.5	59.7	66.5	72.2	94.7
DAILY_MOBILITY_STUDY_WORK	7623	59.859	8.46	13.5	53.1	61.5	66.9	79.4
UNIVERSITY_PRESENCE_INDEX	7623	0.03	0.283	0	0	0	0	16.5
WORK_COMMUTING_MOBILITY_INDEX	7623	0.802	0.071	0.22	0.77	0.82	0.85	0.96
WORK_COMMUTING_SELF_SUFFICIENCY_INDEX	7623	0.3	0.14	0	0.19	0.28	0.39	0.83
PUBLIC_MOBILITY	7623	11.523	4.773	0	8.5	10.7	13.8	50.9
SLOW_MOBILITY	7623	16.729	7.954	0	11.1	15.5	20.9	75.2
EURO_5_AND_6_CARS_PERCENT	7614	9.999	4.058	0	6.6	10.2	12.9	62
POTABLE_WATER_PC	7623	90.75	74.563	0	61.6	75.6	98	2850.7
URBAN_AREAS_COMPACTNESS_INDEX	7511	67.779	23.304	9.9	48.7	69.3	89	100
URBAN_LANDSCAPE_FRAGMENTATION_INDEX	7623	7.976	7.316	0	2.5	5.9	11.6	66.4
OVERCROWDED_POPULATION_PERCENT	7623	1.027	1.009	0	0.4	0.8	1.4	16.7
CONSUMED_SOIL_PC	7623	819.983	808.544	55.4	399.95	614.8	960.75	17097.4
HOTELS_STRUCTURES	84273	4.164	25.031	0	0	1	2	1333
HOTELS_BEDS	85501	282.51	2026.167	0	0	16	103	125079
ADDITIONAL_FACILITIES_STRUCTURES	84273	20.486	250.75	0	1	3	9	19984
ADDITIONAL_FACILITIES_BEDS	85501	347.217	2313.933	0	6	29	114	131427
TOTAL_HOTELS_FACILITIES_STRUCTURES	84273	24.65	265.407	0	1	4	11	21310
TOTAL_HOTELS_FACILITIES_BEDS	85501	629.727	3881.139	0	14	58	244	256400
RECEPTIVITY_INDEX	85362	0.147	0.478	0	0.006	0.021	0.08	10.952
RECEPTIVE_DENSITY_INDEX	85501	18.709	97.766	0	0.683	2.664	8.501	4473.654
MUNICIPAL_ELECTIONS_TURNOUT	16912	0.669	0.109	0.004	0.606	0.686	0.744	1
MUNICIPAL_ELECTIONS_CAMERA_TURNOUT	23040	0.71	0.09	0.176	0.658	0.721	0.778	1.45
POLITICAL_ELECTIONS_SENATO_TURNOUT	22605	0.707	0.09	0.165	0.656	0.719	0.775	1.323
REGIONAL_ELECTIONS_TURNOUT	18480	0.614	0.131	0.029	0.532	0.633	0.712	0.98

Descriptive statistics for the provincial dataset

	COUNT	MEAN	STD	MIN	25%	50%	75%	MAX
GDP_CONST_P_2015	1391	1.6E+10	2.53E+10	1.56E+09	5.92E+09	9.38E+09	1.64E+10	2.11E+11
GDP_CURR_P	1391	1.62E+10	2.56E+10	1.61E+09	5.95E+09	9.42E+09	1.66E+10	2.29E+11
GDP_PPS	1391	1.61E+10	2.55E+10	1.57E+09	5.88E+09	9.38E+09	1.64E+10	2.37E+11
GVA_CONST_P_2015	1391	1.44E+10	2.28E+10	1.41E+09	5.33E+09	8.44E+09	1.48E+10	1.89E+11
GVA_CURR_P	1391	1.46E+10	2.3E+10	1.45E+09	5.35E+09	8.47E+09	1.49E+10	2.07E+11
GVA_PPS	1391	1.45E+10	2.3E+10	1.41E+09	5.29E+09	8.44E+09	1.48E+10	2.15E+11
GDP_PC_CONST_P_2015	1391	25630.14	6916.396	14023.65	19176.79	25611.9	30406.95	48472
GDP_PC_CURR_P	1391	25940.33	7095.222	13930.2	19610.9	25862.55	30551.33	52640.14
GDP_PC_PPS	1391	25819.78	7183.065	13634.21	19605.04	25623.2	30272.23	54530.04
GDP_PE_CONST_P_2015	1391	63349.03	8176.486	45321.5	56732.95	63738.76	69310.91	88837.57
GDP_PE_CURR_P	1391	64097.18	8497.313	47061.91	57552.05	64014.61	70109.44	92353.84
GDP_PE_PPS	1391	63781.57	8885.176	46107.93	57018.12	63674.14	69550.18	95686.38
GDP_G	1391	0.002	0.041	-0.206	-0.017	0.005	0.024	0.192
GVA_CONST_P_2015_A	1177	3.12E+08	2.21E+08	16490000	1.33E+08	2.71E+08	4.32E+08	1.16E+09
GVA_CONST_P_2015_B_E	1177	2.76E+09	3.79E+09	-1.3E+08	8.02E+08	1.46E+09	3.04E+09	3.28E+10
GVA_CONST_P_2015_F	1177	6.77E+08	8.67E+08	89000000	2.78E+08	4.27E+08	7.55E+08	7.97E+09
GVA_CONST_P_2015_G_J	1177	3.5E+09	6.52E+09	2.37E+08	1.19E+09	1.8E+09	3.07E+09	5.72E+10
GVA_CONST_P_2015_K_N	1177	4.06E+09	7.65E+09	3.44E+08	1.36E+09	2.22E+09	3.94E+09	6.79E+10
GVA_CONST_P_2015_O_U	1177	3E+09	4.37E+09	4.6E+08	1.27E+09	1.89E+09	3.23E+09	3.82E+10
GVA_CURR_P_A	1177	3.1E+08	2.2E+08	14800000	1.3E+08	2.69E+08	4.3E+08	1.19E+09
GVA_CURR_P_B_E	1177	2.74E+09	3.77E+09	-1.3E+08	8.02E+08	1.45E+09	3.02E+09	3.19E+10
GVA_CURR_P_F	1177	6.71E+08	8.56E+08	90700000	2.75E+08	4.27E+08	7.51E+08	7.57E+09
GVA_CURR_P_G_J	1177	3.51E+09	6.55E+09	2.39E+08	1.19E+09	1.79E+09	3.13E+09	5.91E+10
GVA_CURR_P_K_N	1177	4.07E+09	7.68E+09	3.36E+08	1.36E+09	2.22E+09	3.97E+09	7.04E+10
GVA_CURR_P_O_U	1177	3.01E+09	4.38E+09	4.83E+08	1.25E+09	1.9E+09	3.28E+09	3.81E+10
GVA_PPS_A	1177	3.06E+08	2.18E+08	14490000	1.29E+08	2.66E+08	4.24E+08	1.18E+09
GVA_PPS_B_E	1177	2.71E+09	3.72E+09	-1.3E+08	7.98E+08	1.44E+09	2.97E+09	3.18E+10
GVA_PPS_F	1177	6.63E+08	8.45E+08	90290000	2.73E+08	4.21E+08	7.38E+08	7.44E+09
GVA_PPS_G_J	1177	3.47E+09	6.48E+09	2.31E+08	1.18E+09	1.78E+09	3.09E+09	5.93E+10
GVA_PPS_K_N	1177	4.02E+09	7.59E+09	3.32E+08	1.35E+09	2.21E+09	3.89E+09	7.07E+10
GVA_PPS_O_U	1177	2.97E+09	4.33E+09	4.87E+08	1.24E+09	1.88E+09	3.24E+09	3.83E+10
GDP_PC_GROWTH_CONST_P_2015	1391	0.004	0.041	-0.206	-0.016	0.006	0.026	0.197
GDP_PC_GROWTH_CURRENT_P	1391	0.016	0.041	-0.191	-0.005	0.016	0.036	0.212
GDP_PC_GROWTH_PPS	1391	0.021	0.045	-0.187	-0.005	0.021	0.042	0.217
GVA_CONST_P_2015_PC	1391	23160.76	6754.182	12702.84	17296.84	22991.48	27296.21	58615.6
GVA_CURR_P_PC	1391	23446.66	6914.456	12537.5	17607.22	23248.92	27448.22	64169.92
GVA_PPS_PC	1391	23337.68	6983.888	12271.1	17617.89	23061.34	27246.12	66473.75
GVA_CONST_P_2015_A_PC	1177	704.688	445.122	56.615	373.511	639.358	939.705	2240.701
GVA_CONST_P_2015_B_E_PC	1177	4651.373	2665.785	-326.261	2069.206	4570.688	6527.894	12663.51
GVA_CONST_P_2015_F_PC	1177	1176.468	365.748	474.059	930.319	1136.141	1368.185	2975.138
GVA_CONST_P_2015_G_J_PC	1177	5235.65	2000.68	2390.006	3930.222	4950.452	6105.641	17643.59
GVA_CONST_P_2015_K_N_PC	1177	6148.682	2271.786	2810.976	4282.862	6142.155	7312.57	20847.62
GVA_CONST_P_2015_O_U_PC	1177	5100.24	1104.579	3453.069	4419.238	4825.133	5480.46	10223.37
GVA_PC_GROWTH_PPS	1284	0.021	0.044	-0.188	-0.006	0.02	0.043	0.217

**GVA_PC_GROWTH_PPS_A**	1070	0	0.082	-0.532	-0.047	-0.004	0.042	0.357
**GVA_PC_GROWTH_PPS_B_E**	1070	-0.017	0.289	-8.948	-0.045	0.001	0.036	0.857
**GVA_PC_GROWTH_PPS_F**	1070	-0.03	0.08	-0.48	-0.079	-0.032	0.015	1.145
**GVA_PC_GROWTH_PPS_G_J**	1070	-0.009	0.066	-0.399	-0.028	0.005	0.028	0.368
**GVA_PC_GROWTH_PPS_K_N**	1070	0.004	0.031	-0.304	-0.011	0.004	0.018	0.23
**GVA_PC_GROWTH_PPS_O_U**	1070	-0.007	0.025	-0.23	-0.02	-0.005	0.007	0.09
**GVA_A_GROWTH_PPS**	1177	0.029	0.136	-0.529	-0.029	0.015	0.067	1.379
**GVA_B_E_GROWTH_PPS**	1177	0.005	0.282	-9.077	-0.029	0.017	0.054	0.894
**GVA_F_GROWTH_PPS**	1177	-0.019	0.086	-0.481	-0.073	-0.019	0.029	1.173
**GVA_G_J_GROWTH_PPS**	1177	0.005	0.068	-0.387	-0.021	0.015	0.043	0.384
**GVA_K_N_GROWTH_PPS**	1177	0.016	0.037	-0.303	-0.002	0.014	0.03	0.252
**GVA_O_U_GROWTH_PPS**	1177	0.006	0.034	-0.144	-0.012	0.005	0.02	0.289
**EMPLOYMENT_SUM**	1391	232922.1	295039	28380	94200	145520	253950	2203100
**EMPLOYMENT_A**	1177	8631.436	6847.807	400	3600	6900	11100	37900
**EMPLOYMENT_B_E**	1177	39999.07	42330.61	3400	13200	26600	45500	262400
**EMPLOYMENT_F**	1177	15293.8	16081.69	2600	6900	10500	18300	134900
**EMPLOYMENT_G_J**	1177	64705.95	89313.33	6600	27000	37800	64500	670600
**EMPLOYMENT_K_N**	1177	35308.92	64070.96	3200	11300	19400	34100	524400
**EMPLOYMENT_O_U**	1177	68325.15	93161.7	9100	29800	41500	77300	822100
**COOPERATIVE_SOCIETIES_WEIGHT**	1037	281349.9	479550.8	25758.12	90824.84	151005.2	295405.3	4148372
**INSTITUTIONAL_QUALITY_INDEX**	1000	0.587	0.253	0	0.39	0.669	0.787	1
**BIRTH_RATE_PER_THOUSAND**	1352	7.514	1.191	4.4	6.675	7.4	8.4	11.3
**AVERAGE_CHILDREN_PER_WOMAN**	1352	1.303	0.125	0.89	1.22	1.3	1.39	1.72
**CHILDBEARING_MEAN_AGE**	1352	31.774	0.617	30.1	31.4	31.8	32.2	33.4
**MORTALITY_RATE_PER_THOUSAND**	1352	11.326	1.783	7.3	10	11.2	12.5	18.9
**LIFE_EXPECTANCY_AT_BIRTH**	1352	82.432	0.86	79.5	81.8	82.4	83.1	84.5
**LIFE_EXPECTANCY_AT_65**	1352	20.501	0.631	17.7	20.1	20.5	20.9	22.1
**NATURAL_GROWTH_PER_THOUSAND**	1352	-3.814	2.768	-13.4	-5.7	-3.7	-1.8	2.6
**INTERNAL_NET_MIGRATION_PER_THOUSAND**	1352	-0.267	2.691	-10.7	-2.025	0.2	1.6	6.9
**EXTERNAL_NET_MIGRATION_PER_THOUSAND**	1352	2.13	1.941	-4.2	0.7	1.9	3.3	9.8
**TOTAL_NET_MIGRATION_PER_THOUSAND**	1352	1.302	4.185	-24.6	-1	1.5	3.8	35.7
**OLD_AGE_DEPENDENCY_RATIO**	1352	36.034	5.277	21.3	32.375	35.7	39.5	49.6
**STRUCTURAL_DEPENDENCE_INDEX**	1352	56.388	4.32	42.7	53.3	56.2	59.325	67.6
**POPULATION_0_14_YEARS_PERCENT**	1352	13.038	1.289	9.7	12.1	13	13.9	17.5
**POPULATION_15_64_YEARS_PERCENT**	1352	63.993	1.764	59.7	62.775	64	65.225	70.1
**POPULATION_65_PLUS_YEARS_PERCENT**	1352	22.971	2.765	14.5	21.1	22.9	24.8	29.7
**OLD_AGE_INDEX_PERCENT**	1352	179.713	37.719	82.5	152.575	176.7	202.825	293.5
**AVERAGE_POPULATION_AGE**	1352	45.355	1.96	39	44	45.4	46.7	50
**PM_10**	706	23.739	6.108	0	19.5	22.9	28.6	35
**OZONE**	660	28.952	25.235	0	5	25	48.75	155
**NITROGEN_DIOXIDE**	688	26.66	9.469	0	21	26	32.425	59
**DIFFERENTIATED_WASTE**	714	0.839	4.325	0	0.394	0.582	0.677	75.7
**PRODUCED_WASTE**	720	526.952	94.414	0	464.2	510.5	581.983	824.054
**TREES_PER_100_INHABITANTS**	595	21.3	21.567	0	10.2	16.8	25.2	205.508
**LAND_USE_EFFICIENCY_INDEX**	721	5.806	2.283	0	3.9	6	7.6	10
**DAYS_WITHOUT_RAIN**	105	27.571	12.403	13	20	23	33	81.5
**VEHICLE_FLEET**	105	477250.8	492113.3	0	208766	318983	570900	3499111
**LOCAL_PUBLIC_TRANSPORT_EXPENDITURE**	104	28280458	1.34E+08	0	632658.9	2744613	6757537	1.17E+09

LOCAL_PUBLIC_TRANSPORT_EXPENDITURE_PC	104	247735.5	274862.5	0	44502.95	163006.8	310762.8	1629022
BIKE_PATHS	196	8.189	8.526	0	1.684	5.124	12.672	46.501
ROADS_INFRASTRUCTURE_EXPENDITURE	105	55439603	62505812	2400641	23496046	39611537	56797163	4.2E+08
CW	105	15330.9	17685.21	355.854	4695.584	9391.268	19808.02	108630.6
VEHICLE_FLEET_UNITS	1177	467285.2	482126.5	0	204932	323261	556592	3732839
RAILWAY_STATIONS_UNITS	856	19.987	14.951	0	10	16	26	98
AIRPORTS_UNITS	1177	0.405	0.533	0	0	0	1	2
AIRCRAFT_TAKEOFFS_LANDINGS_TOTAL	1166	12171.62	41441.51	0	0	0	2967.75	379039
PASSENGERS_AIRPORTS	1166	1400779	5023304	0	0	0	216358.3	49206708
CARGO_AIRPORTS_TONS	1166	8844.36	53111.38	0	0	0	16	589719
INTERNATIONAL_PASSENGERS_AIRPORTS	1166	868865.7	3617088	0	0	0	81755.5	37970723
INTERNATIONAL_FLIGHTS_TAKEOFFS_LANDINGS	1166	7221.219	28013.75	0	0	0	975.25	243039
SHIPS_PORTS_UNITS	1177	3860.713	14753.44	0	0	0	1067	133051
SHIPS_PORTS_TONNAGE	1177	23486716	60683963	0	0	0	7092155	3.97E+08
GOODS_PORTS_TONS	1177	4363331	10203680	0	0	0	1557692	60333286
PASSENGERS_PORTS_UNITS	1177	641694.3	2530466	0	0	0	7500	23362212
BROADBAND_CONNECTIVITY_PERCENT	105	63.584	14.576	19.894	54.922	63.102	74.019	93.025
ENERGY_CONSUMPTION_PER_100_INHABITANTS	105	79.517	30.732	23.181	56.779	79.7	103.568	156.248
BROADBAND_PENETRATION_PERCENT	105	5.8	6.679	0.189	1.586	3.914	7.063	40.352
HOTELS_UP_TO_24_ROOMS	636	169.344	314.101	7	53.75	88.5	172	3104
HOTELS_25_99_ROOMS	636	126.775	204.137	5	30	63	132	1486
HOTELS_OVER_100_ROOMS	636	13.527	22.433	0	1	7	15	158
TOTAL_HOTEL_ESTABLISHMENTS	636	309.646	488.422	14	91	171.5	325.5	4062
WORLD_ARRIVALS_TOTAL	748	1014480	1584864	15417	253147.3	479353	1042790	11416314
WORLD_ARRIVALS_HOTELS	748	748700.7	1224527	11081	176475	343438.5	737855	9238355
WORLD_ARRIVALS_NON_HOTELS	748	265779.2	440279.4	2763	58120.75	133218.5	282677.3	3988461
FOREIGN_ARRIVALS_TOTAL	748	462737.3	1044723	490	48865.25	149340.5	413191	7831864
FOREIGN_ARRIVALS_HOTELS	748	333037.4	778916.5	381	31045	93808	265365	6323449
FOREIGN_ARRIVALS_NON_HOTELS	748	129699.9	307910.3	109	12214.25	38923.5	104504	2965812
ITALY_ARRIVALS_TOTAL	748	551742.5	627872	14927	178292.3	321173	638544.5	3584450
ITALY_ARRIVALS_HOTELS	748	415663.3	518050.6	10210	126044.8	239548	453266	3140737
ITALY_ARRIVALS_NON_HOTELS	748	136079.3	154326.3	2468	39747	83200.5	163250.5	1022649
WORLD_STAYS_TOTAL	748	3494576	5659336	39262	743789.3	1677875	3458276	37951808
WORLD_STAYS_HOTELS	748	2218264	3775940	23820	417110	1020560	2101809	25774246
WORLD_STAYS_NON_HOTELS	748	1276312	2384163	5522	197270.5	577316	1312236	22357937
FOREIGN_STAYS_TOTAL	748	3494576	5659336	39262	743789.3	1677875	3458276	37951808
FOREIGN_STAYS_HOTELS	748	1020466	2397695	1794	84815.5	294479	827254.8	17913063
FOREIGN_STAYS_NON_HOTELS	748	616798	1671917	224	42592.5	162232	430415	16204617
ITALY_STAYS_TOTAL	748	1857312	2245350	37244	530478	1053735	2272871	12361073
ITALY_STAYS_HOTELS	748	1197798	1663213	21859	292162	671651.5	1258339	11544274
ITALY_STAYS_NON_HOTELS	748	659514	852419.7	4824	139255.3	336378.5	945575.3	6701614
RECEPTIVE_DENSITY_INDEX	735	39.136	56.769	1.998	12.438	22.163	42.151	406.671
RECEPTIVITY_INDEX	735	0.194	0.196	0.009	0.065	0.135	0.245	1.05
TOURIST_INTENSITY_INDEX	748	7.258	9.957	0.25	1.766	3.817	7.995	64.526
MUSEUMS_VISITORS	897	502963.9	2148642	0	15775	42841	150765	24490692

**MUSEUMS_GROSS_REVENUE_SERVICES**	897	1755148	8051024	0	6562	35883	245061.5	86058288

Top 3 and bottom 3 municipalities

	TOP 1	TOP 2	TOP 3	BOTTOM 1	BOTTOM 2	BOTTOM 3
**INCOME_PC**	Basiglio	Cusago	Galliate Lombardo	Cavargna	Val Rezzo	Valle Cannobina
**BANK_BRANCHES**	Roma	Milano	Torino	Abetone Cutigliano	Accadia	Accettura
**DEPOSITS**	Milano	Roma	Torino	Abbadia Lariana	Abbasanta	Abetone Cutigliano
**LOANS**	Milano	Roma	Torino	Abbadia Lariana	Abbasanta	Abetone Cutigliano
**EMPLOYEES_B**	San Donato Milanese	Roma	Ravenna	Acerra	Agliana	Airola
**EMPLOYEES_C**	Milano	Roma	Torino	Elva	Fuipiano Valle Imagna	Micigliano
**EMPLOYEES_D**	Roma	Milano	Torino	Airola	Alife	Almese
**EMPLOYEES_E**	Roma	Milano	Napoli	Albiolo	Alfonsine	Almese
**EMPLOYEES_F**	Roma	Milano	Torino	Rosazza	VialfrÃ¨	Langosco
**EMPLOYEES_G**	Roma	Milano	Torino	Oliva Gessi	Roaschia	Castelnuovo di Ceva
**EMPLOYEES_H**	Roma	Milano	Genova	Borgo Lares	Montescheno	Provvidenti
**EMPLOYEES_I**	Roma	Milano	Torino	Duno	Turania	Agnana Calabra
**EMPLOYEES_J**	Roma	Milano	Torino	Alluvioni Piovera	Ardara	Barbaresco
**EMPLOYEES_K**	Milano	Roma	Torino	Azzone	Bonvicino	Casanova Lerrone
**EMPLOYEES_L**	Milano	Roma	Torino	Acceglio	Agosta	Ales
**EMPLOYEES_M**	Milano	Roma	Torino	Ospitale di Cadore	Intragna	Castel Vittorio
**EMPLOYEES_N**	Roma	Milano	Torino	Fabbrica Curone	Petrella Salto	Rassa
**EMPLOYEES_P**	Roma	Milano	Torino	Bolotana	Bubbio	Capriano del Colle
**EMPLOYEES_Q**	Roma	Milano	Torino	Greggio	Saliceto	Avise
**EMPLOYEES_R**	Roma	Milano	Napoli	Alcara li Fusi	Baranello	Casalnuovo Monterotaro
**EMPLOYEES_S**	Roma	Milano	Torino	Civo	Formazza	Parodi Ligure
**EMPLOYEES_TOTAL**	Roma	Milano	Torino	Cervatto	Pedesina	Ingria
**INTERNAL_FOREIGNERS_MIGRATORY_BALANCE**	Prato	Genova	Cinisello Balsamo	Milano	Roma	Napoli
**ITALIAN_CITIZENSHIP_ACQUISITIONS**	Torino	Roma	Milano	Abriola	Acceglio	Accettura
**FOREIGNERS_CENSUS_BALANCE**	Roma	Torino	Genova	Napoli	Corigliano-Rossano	Messina
**UNIVERSITY_ENROLLEES_BY_RESIDENCE**	Roma	Milano	Napoli	Ailoche	Albaredo per San Marco	Anterivo
**ITALIAN_UNIVERSITY_ENROLLEES_BY_RESIDENCE**	Roma	Napoli	Milano	Ailoche	Albaredo per San Marco	Anterivo
**UNIVERSITY_ENROLLEES_CHEMICAL_PHARMACEUTICAL_BY_RESIDENCE**	Roma	Napoli	Milano	Abbateggio	Acquafondata	Acquanegra Cremonese
**UNIVERSITY_ENROLLEES_GEO_BIOLOGICAL_BY_RESIDENCE**	Roma	Napoli	Milano	Abbateggio	Acciano	Acquafredda
**UNIVERSITY_ENROLLEES_MEDICAL_BY_RESIDENCE**	Roma	Napoli	Milano	Abbateggio	Acceglio	Acciano
**UNIVERSITY_ENROLLEES_ENGINEERING_BY_RESIDENCE**	Roma	Napoli	Milano	Abbateggio	Acceglio	Acciano
**UNIVERSITY_ENROLLEES_ARCHITECTURE_BY_RESIDENCE**	Roma	Milano	Torino	Abbasanta	Abbateggio	Acceglio
**UNIVERSITY_ENROLLEES_AGRICULTURAL_BY_RESIDENCE**	Roma	Milano	Torino	Abbadia Cerreto	Acquanegra sul Chiese	Acquaro
**UNIVERSITY_ENROLLEES_ECONOMIC_STATISTICAL_BY_RESIDENCE**	Roma	Milano	Napoli	Acquaviva Collecroce	Acquaviva Platani	Adrara San Rocco
**UNIVERSITY_ENROLLEES_POLITICAL_SOCIAL_BY_RESIDENCE**	Roma	Milano	Torino	Abbadia Cerreto	Acciano	Acquafredda
**UNIVERSITY_ENROLLEES_LEGAL_BY_RESIDENCE**	Roma	Napoli	Milano	Acciano	Acquaviva Collecroce	Acquaviva d'Isernia
**UNIVERSITY_ENROLLEES_LITERARY_BY_RESIDENCE**	Roma	Milano	Napoli	Abbateggio	Acciano	Acquaro
**UNIVERSITY_ENROLLEES_LINGUISTIC_BY_RESIDENCE**	Roma	Napoli	Milano	Abriola	Acciano	Accumoli

**UNIVERSITY_ENROLLEES_TEACHING_BY_RESIDENCE**	Roma	Milano	Torino	Abbadia Cerreto	Acciano	Acquafondata
**UNIVERSITY_ENROLLEES_PSYCHOLOGICAL_BY_RESIDENCE**	Roma	Milano	Napoli	Abbadia Cerreto	Abbateggio	Abriola
**UNIVERSITY_ENROLLEES_PHYSICAL_EDUCATION_BY_RESIDENCE**	Roma	Palermo	Napoli	Acerenza	Acquanegra sul Chiese	Acquappesa
**UNIVERSITY_ENROLLEES_DEFENSE_SECURITY_BY_RESIDENCE**	Pozzuoli	Modena	Roma	Abbasanta	Abbiategrasso	Acerra
**LAUREATES_MAN**	Roma	Milano	Napoli	Abetone Cutigliano	Albera Ligure	Alluvioni Piovera
**LAUREATES_FEMALE**	Roma	Milano	Napoli	Abetone Cutigliano	Alluvioni Piovera	Alpago
**PUBLIC_CARE_INSTITUTIONS**	Roma	Milano	Torino	Acerra	Acqui Terme	Albenga
**PRIVATE_CARE_INSTITUTIONS_ACCREDITED**	Roma	Catania	Palermo	Abano Terme	Abbadia San Salvatore	Acquapendente
**ACUTE_CARE_BEDS_ORDINARY**	Roma	Milano	Torino	Acerra	Acqui Terme	Albenga
**LONG_STAY_CARE_BEDS_ORDINARY**	Bologna	Roma	Reggio nell'Emilia	Abano Terme	Abbadia San Salvatore	Acireale
**REHABILITATION_CARE_BEDS_ORDINARY**	Roma	Milano	Torino	Abbadia San Salvatore	Acerra	Acquapendente
**CARE_BEDS_ORDINARY_TOTAL**	Roma	Milano	Napoli	Carmagnola	Marino	Mendicino
**EMERGENCY_UNIT_DISTANCE**	Lampedusa e Linosa	Ustica	Ponza	Abano Terme	Abbadia San Salvatore	Acerra
**DEATHS_WOMEN**	Roma	Milano	Torino	Blello	Maccastorna	Moncenisio
**DEATHS_MEN**	Roma	Milano	Torino	Moncenisio	Rocca de' Giorgi	Cervatto
**DEATHS_TOTAL**	Roma	Milano	Torino	Moncenisio	Maccastorna	Rocca de' Giorgi
**URBAN_WASTE_PC**	Monteleone di Spoleto	Lignano Sabbiadoro	Limone sul Garda	Asti	Belvedere di Spinello	Calliano Monferrato
**WASTE_COLLECTION_PERCENT**	Civitella San Paolo	Filacciano	Frasso Sabino	Acquafondata	Asti	Belvedere di Spinello
**PM_25**	Flero	Maclodio	Castel Mella	Atrani	Grantola	Miagliano
**URBAN_GREEN_PC**	Balocco	Momperone	Salasco	Abano Terme	Abbadia Cerreto	Abbadia Lariana
**AGRICULTURAL_LAND_PERCENT**	Baceno	Lozzo di Cadore	Valnegra	Campione d'Italia	Casapinta	Claviere
**HIGH_HYDRAULIC_HAZARD_AREAS_PERCENT**	Poggio Renatico	Santa Maria la Fossa	Vigarano Mainarda	Abbateggio	Abriola	Acate
**POPULATION_HIGH_HYDRAULIC_HAZARD_AREAS**	Rimini	Venezia	Genova	Abbadia San Salvatore	Abbateggio	Abriola
**CONSUMED_SOIL_HIGH_HYDRAULIC_HAZARD_AREAS**	Ferrara	Rimini	Ravenna	Abbadia San Salvatore	Abbateggio	Abriola
**INCIDENCE_POPULATION_NUCLEI_SCATTERED_HOUSES**	Proceno	Mongiardino Ligure	Borgomale	Aicurzio	Albaredo per San Marco	Andalo Valtellino
**HOUSING_DISPERSION_INDEX**	Proceno	Arguello	Levice	Acciano	Acerno	Acquanegra Cremonese
**BUILDING_CONCENTRATION_INDEX**	Berzano di San Pietro	Caines	Castroregio	Sant'Eusanio Forconese	Carapelle Calvisio	Ferruzzano
**BUILDING_EXPANSION_INDEX**	Rognano	Roncaro	Castel Gabbiano	Acquaformosa	Agnosine	Airole
**PRIVATE_MOBILITY**	Cervatto	Pedesina	San Benedetto in Perillis	Capri	Monterosso al Mare	Ventotene
**DAILY_MOBILITY_STUDY_WORK**	Cortaccia sulla strada del vino	Lagnasco	Serralunga d'Alba	Massello	Carrega Ligure	Castelmagno
**UNIVERSITY_PRESENCE_INDEX**	Roma	Milano	Napoli	Abano Terme	Abbadia Cerreto	Abbadia Lariana
**WORK_COMMUTING_MOBILITY_INDEX**	Torre de' Negri	Coreglia Ligure	San Nazzaro Val Cavargna	Carrega Ligure	Castelmagno	Massello
**WORK_COMMUTING_SELF_SUFFICIENCY_INDEX**	Genova	Crotone	Goro	Carrega Ligure	Irma	Macra
**PUBLIC_MOBILITY**	Roviano	Montelapiano	Percile	Argentera	Baradili	Briga Alta
**SLOW_MOBILITY**	Ventotene	Pontechianale	Capri	Callabiana	Carrega Ligure	Drenchia
**EURO_5_AND_6_CARS_PERCENT**	Aosta	Trento	Scandicci	Isasca	Monasterolo Casotto	Provvidenti
**POTABLE_WATER_PC**	Foppolo	RhÃªmes-Notre-Dame	Valsavarenche	Belfiore	Calvisano	Castellucchio
**URBAN_AREAS_COMPACTNESS_INDEX**	Abriola	Accadia	Acquaformosa	Sabaudia	Capaccio Paestum	Sessa Aurunca
**URBAN_LANDSCAPE_FRAGMENTATION_INDEX**	Rognano	Roncaro	Castel Gabbiano	Acquaformosa	Agnosine	Airole
**OVERCROWDED_POPULATION_PERCENT**	Pedesina	Ercolano	Ingria	Abbadia Cerreto	Acciano	Accumoli
**CONSUMED_SOIL_PC**	Briga Alta	Carrega Ligure	Morterone	Portici	Atrani	San Giorgio a Cremano

**HOTELS_STRUCTURES**	Roma	Rimini	Milano	Abbadia Cerreto	Acate	Accadia
**HOTELS_BEDS**	Roma	Rimini	Milano	Abbadia Cerreto	Acate	Accadia
**ADDITIONAL_FACILITIES_STRUCTURES**	Roma	San Michele al Tagliamento	Venezia	Abbadia Cerreto	Acquafondat a	Acquafredda
**ADDITIONAL_FACILITIES_BEDS**	Roma	San Michele al Tagliamento	Cavallino-Treporti	Abbadia Cerreto	Acquafondat a	Acquafredda
**TOTAL_HOTELS_FACILITIES_STRUCTURES**	Roma	San Michele al Tagliamento	Venezia	Abbadia Cerreto	Acquafredda	Acquanegra Cremonese
**TOTAL_HOTELS_FACILITIES_BEDS**	Roma	San Michele al Tagliamento	Rimini	Abbadia Cerreto	Acquafredda	Acquanegra Cremonese
**RECEPTIVITY_INDEX**	Valsavarenche	Lignano Sabbiadoro	RhÃªmes-Notre-Dame	Abbadia Cerreto	Acquafredda	Acquanegra Cremonese
**RECEPTIVE_DENSITY_INDEX**	Lignano Sabbiadoro	Cattolica	Riccione	Abbadia Cerreto	Acquafredda	Acquanegra Cremonese
**MUNICIPAL_ELECTIONS_TURNOUT**	Jenne	Marano Equo	Percile	Castelnuovo di Conza	Roio del Sangro	Rosello
**MUNICIPAL_ELECTIONS_CAMERA_TURNOUT**	Sauze di Cesana	Montescano	Moncenisio	San Luca	Staiti	Terravecchia
**POLITICAL_ELECTIONS_SENATO_TURNOUT**	Montescano	Sauze di Cesana	Moncenisio	San Luca	Staiti	Terravecchia
**REGIONAL_ELECTIONS_TURNOUT**	Moncenisio	Ceresole Reale	Montescano	Castelnuovo di Conza	Carrega Ligure	Santomenna

Top 3 and bottom 3 provinces

	TOP 1	TOP 2	TOP 3	BOTTOM 1	BOTTOM 2	BOTTOM 3
**GDP_CONST_P_2015**	Milano	Roma	Torino	Isernia	Vibo Valentia	Enna
**GDP_CURR_P**	Milano	Roma	Torino	Isernia	Vibo Valentia	Enna
**GDP_PPS**	Milano	Roma	Torino	Isernia	Vibo Valentia	Enna
**GVA_CONST_P_2015**	Milano	Roma	Torino	Isernia	Vibo Valentia	Enna
**GVA_CURR_P**	Milano	Roma	Torino	Isernia	Vibo Valentia	Enna
**GVA_PPS**	Milano	Roma	Torino	Isernia	Vibo Valentia	Enna
**GDP_PC_CONST_P_2015**	Milano	Bolzano/Bozen	Bologna	Agrigento	Sud Sardegna	Vibo Valentia
**GDP_PC_CURR_P**	Milano	Bolzano/Bozen	Bologna	Agrigento	Sud Sardegna	Vibo Valentia
**GDP_PC_PPS**	Milano	Bolzano/Bozen	Bologna	Agrigento	Sud Sardegna	Vibo Valentia
**GDP_PE_CONST_P_2015**	Milano	Bolzano/Bozen	Cremona	Vibo Valentia	Cosenza	Barletta-Andria-Trani
**GDP_PE_CURR_P**	Milano	Bolzano/Bozen	Cremona	Vibo Valentia	Cosenza	Barletta-Andria-Trani
**GDP_PE_PPS**	Milano	Bolzano/Bozen	Cremona	Vibo Valentia	Cosenza	Barletta-Andria-Trani
**GDP_G**	Bolzano/Bozen	Bologna	Pordenone	Savona	Enna	Caltanissetta
**GVA_CONST_P_2015_A**	Bolzano/Bozen	Verona	Foggia	Trieste	Verbano-Cusio-Ossola	Prato
**GVA_CONST_P_2015_B_E**	Milano	Torino	Roma	Vibo Valentia	Isernia	Enna
**GVA_CONST_P_2015_F**	Milano	Roma	Torino	Vibo Valentia	Enna	Isernia
**GVA_CONST_P_2015_G_J**	Milano	Roma	Torino	Isernia	Enna	Crotone
**GVA_CONST_P_2015_K_N**	Milano	Roma	Torino	Isernia	Vibo Valentia	Crotone
**GVA_CONST_P_2015_O_U**	Roma	Milano	Napoli	Isernia	Fermo	Vibo Valentia
**GVA_CURR_P_A**	Bolzano/Bozen	Foggia	Verona	Trieste	Verbano-Cusio-Ossola	Prato
**GVA_CURR_P_B_E**	Milano	Torino	Roma	Vibo Valentia	Isernia	Enna
**GVA_CURR_P_F**	Milano	Roma	Torino	Vibo Valentia	Enna	Isernia
**GVA_CURR_P_G_J**	Milano	Roma	Torino	Isernia	Enna	Crotone
**GVA_CURR_P_K_N**	Milano	Roma	Torino	Isernia	Vibo Valentia	Crotone
**GVA_CURR_P_O_U**	Roma	Milano	Napoli	Isernia	Fermo	Vibo Valentia
**GVA_PPS_A**	Bolzano/Bozen	Foggia	Verona	Trieste	Verbano-Cusio-Ossola	Prato
**GVA_PPS_B_E**	Milano	Torino	Roma	Vibo Valentia	Isernia	Enna
**GVA_PPS_F**	Milano	Roma	Torino	Vibo Valentia	Enna	Isernia

**GVA_PPS_G_J**	Milano	Roma	Torino	Isernia	Enna	Crotone
**GVA_PPS_K_N**	Milano	Roma	Torino	Isernia	Vibo Valentia	Crotone
**GVA_PPS_O_U**	Roma	Milano	Napoli	Isernia	Fermo	Vibo Valentia
**GDP_PC_GROWTH_CONST_P_2015**	Potenza	Cremona	Pordenone	Ragusa	Savona	Roma
**GDP_PC_GROWTH_CURRENT_P**	Potenza	Pordenone	Cremona	Ragusa	Roma	Fermo
**GDP_PC_GROWTH_PPS**	Potenza	Cremona	Pordenone	Ragusa	Roma	Fermo
**GVA_CONST_P_2015_PC**	Milano	Bolzano/Bozen	Bologna	Agrigento	Sud Sardegna	Vibo Valentia
**GVA_CURR_P_PC**	Milano	Bolzano/Bozen	Bologna	Agrigento	Sud Sardegna	Vibo Valentia
**GVA_PPS_PC**	Milano	Bolzano/Bozen	Bologna	Agrigento	Sud Sardegna	Vibo Valentia
**GVA_CONST_P_2015_A_PC**	Bolzano/Bozen	Pistoia	Mantova	Monza e della Brianza	Genova	Trieste
**GVA_CONST_P_2015_B_E_PC**	Modena	Vicenza	Reggio nell'Emilia	Reggio Calabria	Cosenza	Vibo Valentia
**GVA_CONST_P_2015_F_PC**	Bolzano/Bozen	Valle d'Aosta/Vallée d'Aoste	Milano	Palermo	Trapani	Agrigento
**GVA_CONST_P_2015_G_J_PC**	Milano	Bolzano/Bozen	Roma	Enna	Agrigento	Caltanissetta
**GVA_CONST_P_2015_K_N_PC**	Milano	Roma	Bologna	Vibo Valentia	Sud Sardegna	Agrigento
**GVA_CONST_P_2015_O_U_PC**	Valle d'Aosta/Vallée d'Aoste	Roma	Bolzano/Bozen	Barletta-Andria-Trani	Fermo	Lecco
**GVA_PC_GROWTH_PPS**	Piacenza	Potenza	Matera	Ragusa	Crotone	Fermo
**GVA_PC_GROWTH_PPS_A**	Vibo Valentia	Barletta-Andria-Trani	Crotone	Milano	La Spezia	Savona
**GVA_PC_GROWTH_PPS_B_E**	Trieste	Genova	Massa-Carrara	Siracusa	Crotone	Milano
**GVA_PC_GROWTH_PPS_F**	Ascoli Piceno	Bolzano/Bozen	Pordenone	Ravenna	Vibo Valentia	Agrigento
**GVA_PC_GROWTH_PPS_G_J**	Belluno	Matera	Cremona	Como	Genova	Teramo
**GVA_PC_GROWTH_PPS_K_N**	Imperia	La Spezia	Pisa	Ragusa	Reggio Calabria	Roma
**GVA_PC_GROWTH_PPS_O_U**	Trieste	Imperia	Sud Sardegna	Milano	Campobasso	Sondrio
**GVA_A_GROWTH_PPS**	Milano	Barletta-Andria-Trani	Vibo Valentia	La Spezia	Trieste	Savona
**GVA_B_E_GROWTH_PPS**	Milano	Trieste	Vercelli	Siracusa	Crotone	Nuoro
**GVA_F_GROWTH_PPS**	Milano	Ascoli Piceno	Bolzano/Bozen	Agrigento	Ravenna	Vibo Valentia
**GVA_G_J_GROWTH_PPS**	Milano	Belluno	Monza e della Brianza	Genova	Savona	Teramo
**GVA_K_N_GROWTH_PPS**	Milano	Pordenone	La Spezia	Reggio Calabria	Biella	Enna
**GVA_O_U_GROWTH_PPS**	Milano	La Spezia	Bolzano/Bozen	Campobasso	Napoli	Matera
**EMPLOYMENT_SUM**	Roma	Milano	Torino	Isernia	Enna	Vibo Valentia
**EMPLOYMENT_A**	Cosenza	Bari	Reggio Calabria	Prato	Trieste	Verbano-Cusio-Ossola
**EMPLOYMENT_B_E**	Milano	Torino	Brescia	Vibo Valentia	Enna	Oristano
**EMPLOYMENT_F**	Roma	Milano	Napoli	Isernia	Enna	Oristano
**EMPLOYMENT_G_J**	Roma	Milano	Napoli	Isernia	Enna	Rieti
**EMPLOYMENT_K_N**	Milano	Roma	Torino	Isernia	Vibo Valentia	Enna
**EMPLOYMENT_O_U**	Roma	Milano	Napoli	Isernia	Fermo	Vibo Valentia
**COOPERATIVE_SOCIETIES_WEIGHT**	Milano	Roma	Torino	Isernia	Rieti	Enna
**INSTITUTIONAL_QUALITY_INDEX**	Trieste	Trento	Gorizia	Vibo Valentia	Crotone	Caltanissetta
**BIRTH_RATE_PER_THOUSAND**	Catania	Napoli	Caserta	Oristano	Sud Sardegna	Biella
**AVERAGE_CHILDREN_PER_WOMAN**	Trento	Reggio nell'Emilia	Cuneo	Oristano	Sud Sardegna	Cagliari
**CHILDBEARING_MEAN_AGE**	Oristano	Nuoro	Cagliari	Siracusa	Crotone	Catania
**MORTALITY_RATE_PER_THOUSAND**	Alessandria	Trieste	Genova	Barletta-Andria-Trani	Cagliari	Caserta
**LIFE_EXPECTANCY_AT_BIRTH**	Firenze	Treviso	Trento	Napoli	Caserta	Caltanissetta
**LIFE_EXPECTANCY_AT_65**	Trento	Treviso	Firenze	Napoli	Caserta	Caltanissetta
**NATURAL_GROWTH_PER_THOUSAND**	Napoli	Caserta	Barletta-Andria-Trani	Alessandria	Savona	Trieste

**INTERNAL_NET_MIGRATION_PER_THOUSAND**	Bologna	Parma	Rimini	Vibo Valentia	Caltanissetta	Crotone
**EXTERNAL_NET_MIGRATION_PER_THOUSAND**	Milano	Parma	Ragusa	Sud Sardegna	Palermo	Enna
**TOTAL_NET_MIGRATION_PER_THOUSAND**	Parma	Bologna	Milano	Enna	Vibo Valentia	Caltanissetta
**OLD_AGE_DEPENDENCY_RATIO**	Savona	Trieste	Genova	Caserta	Napoli	Barletta-Andria-Trani
**STRUCTURAL_DEPENDENCE_INDEX**	Savona	Genova	Trieste	Caserta	Cagliari	Napoli
**POPULATION_0_14_YEARS_PERCENT**	Napoli	Caserta	Crotone	Oristano	Sud Sardegna	Ferrara
**POPULATION_15_64_YEARS_PERCENT**	Caserta	Cagliari	Napoli	Savona	Genova	Trieste
**POPULATION_65_PLUS_YEARS_PERCENT**	Savona	Trieste	Genova	Caserta	Napoli	Barletta-Andria-Trani
**OLD_AGE_INDEX_PERCENT**	Savona	Trieste	Genova	Napoli	Caserta	Barletta-Andria-Trani
**AVERAGE_POPULATION_AGE**	Savona	Trieste	Genova	Napoli	Caserta	Crotone
**PM_10**	Padova	Cremona	Alessandria	Isernia	Matera	Nuoro
**OZONE**	Bergamo	Lecco	Lodi	Foggia	Isernia	Massa-Carrara
**NITROGEN_DIOXIDE**	Monza e della Brianza	Torino	Milano	Matera	Potenza	Enna
**DIFFERENTIATED_WASTE**	Macerata	Monza e della Brianza	Pesaro e Urbino	Catania	Crotone	Palermo
**PRODUCED_WASTE**	Massa-Carrara	Piacenza	Ravenna	Nuoro	Potenza	Reggio Calabria
**TREES_PER_100_INHABITANTS**	Modena	Cuneo	Brescia	Ancona	Campobasso	Latina
**LAND_USE_EFFICIENCY_INDEX**	Milano	Napoli	Bolzano/Bozen	Ragusa	Brindisi	Enna
**DAYS_WITHOUT_RAIN**	Cagliari	Sud Sardegna	Ragusa	Genova	Cuneo	Treviso
**VEHICLE_FLEET**	Roma	Milano	Napoli	Sud Sardegna	Isernia	Gorizia
**LOCAL_PUBLIC_TRANSPORT_EXPENDITURE**	Milano	Roma	Napoli	Sud Sardegna	Gorizia	Caserta
**LOCAL_PUBLIC_TRANSPORT_EXPENDITURE_PC**	Milano	Roma	Frosinone	Sud Sardegna	Caserta	Trieste
**BIKE_PATHS**	Reggio nell'Emilia	Cremona	Mantova	Chieti	Potenza	Vibo Valentia
**ROADS_INFRASTRUCTURE_EXPENDITURE**	Roma	Milano	Napoli	Caltanissetta	Enna	Crotone
**CW**	Cuneo	Trento	Torino	Ragusa	Caltanissetta	Barletta-Andria-Trani
**VEHICLE_FLEET_UNITS**	Roma	Milano	Napoli	Isernia	Sud Sardegna	Gorizia
**RAILWAY_STATIONS_UNITS**	Roma	Milano	Torino	Fermo	Barletta-Andria-Trani	Nuoro
**AIRPORTS_UNITS**	Roma	Trapani	Sassari	Alessandria	Arezzo	Ascoli Piceno
**AIRCRAFT_TAKEOFFS_LANDINGS_TOTAL**	Roma	Milano	Varese	Alessandria	Arezzo	Ascoli Piceno
**PASSENGERS_AIRPORTS**	Roma	Milano	Varese	Alessandria	Arezzo	Ascoli Piceno
**CARGO_AIRPORTS_TONS**	Varese	Roma	Milano	Alessandria	Arezzo	Ascoli Piceno
**INTERNATIONAL_PASSENGERS_AIRPORTS**	Roma	Varese	Milano	Alessandria	Arezzo	Ascoli Piceno
**INTERNATIONAL_FLIGHTS_TAKEOFFS_LANDINGS**	Roma	Milano	Varese	Alessandria	Arezzo	Ascoli Piceno
**SHIPS_PORTS_UNITS**	Napoli	Messina	Reggio Calabria	Alessandria	Arezzo	Asti
**SHIPS_PORTS_TONNAGE**	Livorno	Reggio Calabria	Messina	Alessandria	Arezzo	Asti
**GOODS_PORTS_TONS**	Trieste	Genova	Cagliari	Alessandria	Arezzo	Ascoli Piceno
**PASSENGERS_PORTS_UNITS**	Napoli	Messina	Livorno	Alessandria	Arezzo	Ascoli Piceno
**BROADBAND_CONNECTIVITY_PERCENT**	Siracusa	Trieste	Taranto	Isernia	Crotone	L'Aquila
**ENERGY_CONSUMPTION_PER_100_INHABITANTS**	Vicenza	Pavia	Padova	Sud Sardegna	Nuoro	Sassari
**BROADBAND_PENETRATION_PERCENT**	Milano	Cagliari	Bologna	Enna	Massa-Carrara	Gorizia
**HOTELS_UP_TO_24_ROOMS**	Bolzano/Bozen	Roma	Rimini	Enna	Prato	Caltanissetta
**HOTELS_25_99_ROOMS**	Rimini	Bolzano/Bozen	Trento	Caltanissetta	Biella	Enna
**HOTELS_OVER_100_ROOMS**	Roma	Milano	Sassari	Asti	Barletta-Andria-Trani	Benevento
**TOTAL_HOTEL_ESTABLISHMENTS**	Bolzano/Bozen	Rimini	Roma	Enna	Caltanissetta	Isernia

**WORLD_ARRIVALS_TOTAL**	Roma	Venezia	Bolzano/Bozen	Isernia	Rieti	Benevento
**WORLD_ARRIVALS_HOTELS**	Roma	Bolzano/Bozen	Milano	Isernia	Enna	Benevento
**WORLD_ARRIVALS_NON_HOTELS**	Venezia	Verona	Roma	Isernia	Lodi	Rieti
**FOREIGN_ARRIVALS_TOTAL**	Venezia	Roma	Bolzano/Bozen	Isernia	Benevento	Caltanissetta
**FOREIGN_ARRIVALS_HOTELS**	Roma	Venezia	Bolzano/Bozen	Isernia	Benevento	Campobasso
**FOREIGN_ARRIVALS_NON_HOTELS**	Venezia	Verona	Roma	Isernia	Benevento	Avellino
**ITALY_ARRIVALS_TOTAL**	Roma	Rimini	Milano	Isernia	Enna	Rieti
**ITALY_ARRIVALS_HOTELS**	Rimini	Roma	Milano	Enna	Isernia	Benevento
**ITALY_ARRIVALS_NON_HOTELS**	Venezia	Verona	Trento	Lodi	Isernia	Rieti
**WORLD_STAYS_TOTAL**	Venezia	Bolzano/Bozen	Roma	Isernia	Enna	Rieti
**WORLD_STAYS_HOTELS**	Bolzano/Bozen	Roma	Rimini	Enna	Isernia	Benevento
**WORLD_STAYS_NON_HOTELS**	Venezia	Verona	Bolzano/Bozen	Isernia	Rieti	Avellino
**FOREIGN_STAYS_TOTAL**	Venezia	Bolzano/Bozen	Roma	Isernia	Enna	Rieti
**FOREIGN_STAYS_HOTELS**	Bolzano/Bozen	Roma	Venezia	Isernia	Enna	Rieti
**FOREIGN_STAYS_NON_HOTELS**	Venezia	Verona	Bolzano/Bozen	Isernia	Lodi	Frosinone
**ITALY_STAYS_TOTAL**	Rimini	Bolzano/Bozen	Trento	Isernia	Enna	Benevento
**ITALY_STAYS_HOTELS**	Rimini	Bolzano/Bozen	Trento	Enna	Isernia	Benevento
**ITALY_STAYS_NON_HOTELS**	Venezia	Livorno	Grosseto	Isernia	Rieti	Avellino
**RECEPTIVE_DENSITY_INDEX**	Rimini	Venezia	Livorno	Enna	Isernia	Caltanissetta
**RECEPTIVITY_INDEX**	Rimini	Venezia	Grosseto	Monza e della Brianza	Barletta-Andria-Trani	Lodi
**TOURIST_INTENSITY_INDEX**	Bolzano/Bozen	Valle d'Aosta/Vallée d'Aoste	Rimini	Benevento	Avellino	Enna
**MUSEUMS_VISITORS**	Roma	Napoli	Firenze	Pordenone	Verona	Cremona
**MUSEUMS_GROSS_REVENUE_SERVICES**	Roma	Napoli	Firenze	Pordenone	Verona	Cremona
